# Decision support system based on bipolar complex fuzzy Hamy mean operators

**DOI:** 10.1016/j.heliyon.2024.e36461

**Published:** 2024-08-21

**Authors:** Zhuoan Zhao, Abrar Hussain, Nan Zhang, Kifayat Ullah, Shi Yin, Amrullah Awsar, Salah M. El-Bahy

**Affiliations:** a School of Economics and Management, Harbin Engineering University, Harbin, 150000, China; b Department of Mathematics, Riphah International University (Lahore Campus), 54000, Lahore, Pakistan; c College of Humanities and Social Sciences, Hebei Agricultural University, Baoding, 071000, China; d College of Economics and Management, Hebei Agricultural University, Baoding, 071000, China; e Department of Higher Mathematics, Kabul Polytechnic University, Kabul, Afghanistan; f Department of Chemistry, Turabah University College, Taif University, P.O. Box 11099, Taif 21944, Saudi Arabia

**Keywords:** Bipolar complex fuzzy values, Hamy mean, Aggregation operators, Multi-attribute decision-making, Investment Policy

## Abstract

The important feature of the multi-attribute decision-making (MADM) technique is to identify an ideal solution and aggregate collective cognitive fuzzy information of human opinion. To serve this purpose, we explore the concepts of the bipolar complex fuzzy set with positive and negative support terms. A few applications of the Hamy mean (HM) and Dual Hamy mean (DHM) models are also discussed to find out the relationship among input arguments or different preferences. For this, we derive a family of mathematical approaches by incorporating the theory of bipolar complex fuzzy information such as bipolar complex fuzzy Hamy mean (BCFHM), bipolar complex fuzzy weighted Hamy mean (BCFWHM), bipolar complex fuzzy Dual Hamy mean (BCFDHM), and bipolar complex fuzzy weighted Dual Hamy mean (BCFWDHM) operators. Derived mathematical approaches are more applicable and can express the influence of uncertain information due to the involvement of additional parameter values. Based on diagnosed research work and mathematical methodologies, we establish a decision algorithm for the MADM problem to resolve real-life dilemmas. An experimental case study demonstrates the compatibility of derived approaches and evaluates the investment policy's sustainability based on certain parameters. The advantages and consistency of the proposed research work are verified in the comparative study with various existing aggregation operators.

## 1 Introduction

Multi-Attribute Decision-Making (MADM) is a decision-making approach that involves evaluating and comparing alternatives based on multiple criteria or attributes. This method is widely used in various fields such as business, engineering, finance, and public policy, where decisions often involve considering multiple conflicting objectives. The MADM provides a structured framework to systematically analyze, rank, and select alternatives in complex decision environments. In this study, the proposed MADM model offers significant managerial insights by providing a structured framework for evaluating multiple criteria simultaneously. This model enhances decision-making by allowing managers to systematically compare alternatives based on various relevant attributes, thus ensuring a comprehensive analysis. By incorporating qualitative and quantitative factors, the MADM model helps identify the most optimal choice that aligns with organizational goals and constraints. Additionally, the model aids in reducing cognitive biases and subjective judgments, leading to more objective and rational decisions. The experts can leverage this approach to improve strategic planning, resource allocation, and operational efficiency, ultimately driving better outcomes for the organization. The impact of multi-attribute decision-making is far-reaching, influencing decision quality, transparency, and efficiency across various contexts. As decision problems become more complex, the use of MADM techniques becomes increasingly valuable in navigating uncertainties and making well-informed choices. To overcome the impact of the above-discussed challenges, Zadeh [1] diagnosed a feasible theory of fuzzy set (FS) by exploring the concepts of classical set theory. In classical set theory, a membership grade expresses the belongingness of an element to a set with two binary numbers 0 and 1. i.e. {0,1}. But the FS assigns a specific membership to an element of set on a close unit interval [0,1]. Later on, numerous research scholars utilized the theory of FS to resolve different real-life applications, such as the theory of N-soft set [2], mathematical approaches of weighted average and weighted geometric [3], and N-soft topological theory [4]. The Fuzzy logic theory is used to resolve many genuine real-life applications and amplifications such as control systems, pattern recognition, robotics, medical diagnosis, traffic control, environmental modelling, quality control, and human-machine interface. However, there are also many uncertainties in the theory of FS. For example, if a skilful person provides information in the form of a duplet (x,y) where the values of " x" and " y" belong to [0,1] and [−1,0], respectively, it is evident that the theory of FS has been limited. Zhang [5] diagnosed the central idea of BFS to diagnose the solution to the hypothesis mentioned above. The primary advantage of BFS is that it combines two distinct grades into a single structure: the negative membership grade, whose value is found in [−1,0], and the positive membership grade, whose value is found in [0,1]. Since the theory of BFS is more developed than the theory of FS, it has been used by several scientists in a variety of fields. For example, Dombi aggregation operators based on BFSs [6] and the VIKOR method with distance and entropy measures by incorporating the theory of Einstein aggregation operators [7]. Qiyas et al. [8] utilized the theory of confidence levels under considering the system of BCF environments and the decision analysis process.

Ramot et al. [9] deliberated a new theory of complex fuzzy set (CFS) by adding a phase term with a real term and exploring the real plane to a complex plane with unit interval $i=\sqrt{-1}$. Ramot et al. [10] also characterized some fundamental operations under considering the theory of CFSs. Ali et al. [11] anticipated prioritized mathematical approaches and the decision analysis process. Dong et al. [12] presented Hamacher aggregation operators to handle real-life applications and different amplifications. The expert may have encountered information in the form of a duplet ((δ,θ),(ν,φ)) in some circumstances, where (δ,θ)∈[0,1] represented the positive membership grade in the form of Cartesian form and (ν,φ)∈[−1,0] represented the negative membership grade. In such cases, the theory of FSs, BFSs, and CFSs may not have been trustworthy. Mahmood and Ur Rehman [13] diagnosed the principal concept of BCF information in order to diagnosis the solution of the theory mentioned above. The main advantage of BCF information is its superiority over FS, BFS, and CFS theories. Numerous academics have used it in a variety of fields, such as Gwak et al. [14] investigated some hybrid algorithms of the decision-making process based on BCF information.

### 1.1 Literature review

The MADM model significantly enhances decision-making by providing a structured and systematic framework to evaluate multiple often conflicting criteria. This model aids decision-makers by breaking down complex decisions into more manageable parts, allowing for a comprehensive analysis of each alternative based on various attributes. The MADM involves identifying and listing all relevant criteria that impact the decision. The uniqueness of an advanced decision-making technique is that it assesses a reliable and accurate solution from a complicated real-life dilemma. The aggregation operators play an efficient role in the decision analysis to classify the optimal option under considering particular characteristics or attributes. Scientists and mathematicians anticipated different realistic mathematical methodologies based on different fuzzy domains. For instance, An advanced decision-making technique of the TOPSIS method and modified Bonferroni mean operations were established by Ali et al. [15]. Hussain et al. [16] deliberated various mathematical terminologies for investigating unknown degrees of weight criteria or attribute information. A novel approach to Sugeno-Weber aggregations operators was developed by Wang et al. [17]. Hussain et al. [18] deliberated various Dombi aggregation operators for evaluating the performance of the decision algorithm. Özer [19] proposed robust terminologies of prioritized Hamacher aggregation operators. Ali et al. [20] put forward the theory of Heronian mean operators under the system of decision algorithm of the MADM problem. Ali [21] gave new approaches to complex intuitionistic fuzzy theory based on interaction operations. Hussain and Pamucar [22] modified the theory of pythagorean fuzzy rough information using Schweizer-Sklar triangular norms. Seikh and Mandal [23] developed novel approaches based on Dombi operations to resolve an application of the bio-medical waste organization using the SWARA-based PROMETHEE II method. Mandal and Seikh [24] also studied an innovative technique of the MABAC method to evaluate a reliable option for plastic waste enterprises. Hussain et al. [25] evaluated safety tools using croquet integral operators and the q-rung orthopair fuzzy environment. Seikh and Mandal [26] utilized the theory of q-rung orthopair fuzzy information and Archimedean t-norms to derive new mathematical approaches under the system of the decision-making process. Liu et al. [27] deliberated different hybrid mathematical approaches to evaluate the environmental impact of ships using entropy measures and advanced decision analysis processes. Safaeian et al. [28] put forward the theory of incremental discount under the system of fuzzy framework and multi objective optimization techniques. Wang et al. [29] applied a novel theory of 2-tuple linguistic intuitionistic fuzzy information and regret theory to select a suitable design for disassembly. Pouresmaeil et al. [30] established an innovative decision algorithm for coagulation and flocculation developments. Moslem [31] characterized an analytic hierarchy method for the evaluation of sustainable transport under considering spherical fuzzy information. Mahmood et al. [32] developed robust operational laws of Aczel Alsina t-norm and t-conorm under considering BCF information to derive mathematical strategies and decision algorithms for the MADM problem. Hussain et al. [33] resolved a real-life application for a suitable supplier selection with the decision algorithm of the MADM problem. Hussain et al. [34] modified Dombi aggregation operators by incorporating Hamy mean models and t-spherical fuzzy information theory. Sarkar et al. [35] developed an innovative approach of the Sugeno-Weber t-norms and t-conorms to investigate quantum computing. Kumar and Chen [36] interpreted a series of realistic mathematical approaches that take into account an interval-valued intuitionistic fuzzy theory. Farid and Riaz [37] invented a decision algorithm for quantum computing based on q-rung orthopair fuzzy information and Aczel Alsina operations.

Chakraborty and Saha [38] discussed the properties of Bonferroni mean operators for choosing a dominant healthcare waste technology. Farid et al. [39] investigated aggregation operators based on algebraic t-norm and t-conorm for a dynamic decision-making process. Akram et al. [40] investigated Hamacher aggregation operators to classify different types of attributes and assess the finest optimal option. Zheng et al. [41] established an efficient model of mutation samples based on deep learning and programming coding. Cheng et al. [42] improved advanced technology of internet infrastructure of situation-aware dynamic models. Zheng et al. [43] discussed the question-answering method in the natural language processing and pre-training models. Wang et al. [44] introduced novel stability criteria for the Lure system by considering Lyapunov matrices. Hussain et al. [45] employed different recycling models for reducing the impact of waste and useless materials with the help of Schweizer Sklar mathematical models. Xu et al. [46] evaluated suitable transport based on various preferences for oil distribution. Zhou et al. [47] investigated an authentic decision algorithm for photo selection in crowdsensing. Hussain et al. [48] also characterized the theory of Aczel Alsina operators to investigate a reliable electric motor car based on the decision support system. Akram and Martino [49] generated the concepts of arithmetic mean operator based on t-spherical fuzzy soft rough theory. Al-Barakati et al. [50] evaluated dominant renewable energy sources based on the WASPAS method and interval-valued pythagorean fuzzy environments. Akram and Shahzadi [51] investigated realistic mathematical approaches for quantum computing with a hybrid decision support system. Numerous research scholars have developed many reliable mathematical models and methodologies under different fuzzy domains [[52], [53], [54], [55]].

The theory of the HM operator was introduced to describe the relationship among different types of attribute information [56]. Considering the robustness and flexibility of the HM operator, several mathematicians explored the properties of HM operators to interpret different mathematical approaches and methodologies. For example, Ali et al. [57] illustrated a family of new mathematical approaches of the HM operators for handling ambiguous information of human opinion. Hussain et al. [58] investigated Dombi HM operators for resolving complicated real-life applications and numerical examples. Wang et al. [59] modified partitioned Hamy mean operators by exploring the theory of Neutrosophic set and decision-making problems. Hussain et al. [60] interpreted the properties of Hamy mean and Aczel Alsina operators for the assessment of construction materials. Liu et al. [61] anticipated a list of new Hamy mean models for unknown weights and robust decision algorithms of the MADM problem. Garg et al. [62] also presented a series of Hamy mean operators that take into account complex q-rung orthopair fuzzy theory.

### 1.2 Motivations and contributions behind the research work

It is clear that the above-discussed mathematical approaches and strategies have many advantages and the capability to handle ambiguous types of information about human opinion. On the other hand, many mathematical models express correlation among attributes or characteristics, such as symmetric mean, Heronian mean, Bonferroni mean, and Muirhead mean operators. However, the HM operator is more flexible and can provide smooth approximated outcomes during the decision analysis process. To manage the relationship among input arguments, the binomial coefficient plays an effective role in the pairwise relation. The BCF theory is an effective and dominant modification of complex fuzzy sets and bipolar fuzzy theory to manage redundant and ambiguous information of genuine real-life applications. The BCF information covers both positive and negative aspects of membership grades in the shape of a cartesian form of complex values. The bipolar fuzzy theory contains limited information about any object. So, there is a clear chance of losing particular information due to neglecting imaginary parts. The experts face many crucial challenges due to eliminating the imaginary parts from the BCF theory and the loss of important information about real-life applications. That's why the BCF theory is more reliable and evaluates both dimensions in a single term. Motivated by the significance of BCF theory, we developed a massive and dominant research work to tackle redundant and fuzzy information about human opinion. The main contributions and characteristics of this presented research are given below.

a) Demonstrate the flexibility and supremacy of BCF information and its fundamental features by modifying algebraic t-norm and t-conorm in addition, multiplication, scalar multiplication, and power rule.
b) To assess the relationship among different input arguments, we generalize the theory of HM and DHM models to construct an innovative mathematical approach in light of BCF information.
c) Based on BCF information, we derived a family of robust mathematical strategies including BCFHM, BCFWHM, BCFDHM and BCFWDHM operators with feasible properties.
d) To enhance the performance of invented approaches, we established a decision algorithm for the MADM problem in the presence of BCF environments.
e) With the help of numerical examples, we revealed the intensity and worth of diagnosed research work. The influence study and comparison technique with existing approaches are also designed to illustrate the compatibility and effectiveness of the presented research work.

### 1.3 Structure of the article

The outlines of this article are maintained as follows: section 2 explores some primary notions and fundamental preliminaries of BCFSs, HM, and DHM operators. We derived a series of mathematical approaches of the BCF information such as BCFHM and BCFWHM operators in section 3. Section 4 also diagnosed some robust aggregation operators of the BCFDHM and BCFWDHM operators. In section 5, an innovative approach to the MADM problem is used to resolve genuine real-life applications and numerical examples with the help of derived mathematical strategies. Section 6 also illustrates advantages and comparison techniques to analyze the robustness of invented research work. Finally, concluding remarks are presented in section 7.

List of symbols for this article.

**Table:** 

Symbols	Description	Symbols	Description
Q	Source set	ν	Negative membership grade
δ	Positive membership grade	x	Element of the source set
θ	Imaginary term of positive membership grade	φ	Imaginary term of negative membership grade
H	Score function	H	Accuracy function
τ	Bipolar complex fuzzy value	w	Weight vector
A	Alternatives	L	Attributes

## 2 Preliminaries

This section contains primary notions of BFSs, BCFSs, score and accuracy function, and operational laws of algebraic t-norm and t-conorm, HM, and DHM models.

Definition 1 [63] An IFS D is expressed as follows:

D={(x,δ(x),ν(x))|x∈Q}

The mathematical shape of an IFS is expressed with the condition 0≤δ(x)+ν(x)≤1, where δ(x)∈[0,1] and ν(x)∈[0,1] represent the positive membership grade and negative membership grade, respectively. The hesitancy grade of an IFS is denoted by π(x)=1−(δ(x)+ν(x)).

Definition 2 [5] A mathematical shape of BFS D is characterized by a universal set Q as follows:

D={(x,δ(x),ν(x))|x∈Q}

Where δ(x)∈[0,1] and ν(x)∈[−1,0] denote the positive membership grade and the negative membership grade, respectively, and the pair (δ(x),ν(x)) is known as the bipolar fuzzy value.

Definition 3 [13] A mathematical shape of BCFS D is characterized by a universal set Q as follows:

D={(x,δ(x)+iθ(x),ν(x)+iφ(x))|x∈Q}

The symbols δ(x) and ν(x) Indicate the real term of the positive and negative membership grades. While θ(x) and φ(x) represent the imaginary term of positive membership grade and negative membership grade. Where the terms δ(x),θ(x)∈[0,1] and ν(x),φ(x)∈[−1,0]. The bipolar complex fuzzy value (BCFV) is represented by $\tau =\left(\delta_{\tau }\left(x\right)+i\theta_{\tau }\left(x\right),\nu_{\tau }\left(x\right)+i\varphi_{\tau }\left(x\right)\right)$.

Definition 4 [32] If $\tau =\left(\delta_{\tau }\left(x\right)+i\theta_{\tau }\left(x\right),\nu_{\tau }\left(x\right)+i\varphi_{\tau }\left(x\right)\right)$ be a BCFV. The score function H(τ) and accuracy function H(τ) are expressed in the following Eq. (1) and Eq. (2) respectively:

(1) $$H\left(\tau \right)=\frac{1}{4}\left(2+\delta_{\tau }\left(x\right)+\theta_{\tau }\left(x\right)+\nu_{\tau }\left(x\right)+\varphi_{\tau }\left(x\right)\right)$$

and

(2) $$H\left(\tau \right)=\frac{1}{4}\left(\delta_{\tau }\left(x\right)+\theta_{\tau }\left(x\right)+\nu_{\tau }\left(x\right)+\varphi_{\tau }\left(x\right)\right)$$

It is obvious that H(τ)∈[0,1] and H(τ)∈[0,1].

Definition 5 For two BCFVs $\tau =\left(\delta_{\tau }\left(x\right)+i\theta_{\tau }\left(x\right),\nu_{\tau }\left(x\right)+i\varphi_{\tau }\left(x\right)\right)$ and $R=\left(\delta_{R}\left(x\right)+i\theta_{R}\left(x\right),\nu_{R}\left(x\right)+i\varphi_{R}\left(x\right)\right)$. We introduce the order relation among τ and R if and only if:

i. If H(τ)>H(R), then τ proceed to R.
ii. If H(τ)<H(R), then R proceed to τ.
iii. If H(τ)=H(R), Then:
a) H(τ)>H(R), then τ proceed to R.
b) H(τ)<H(R), then R proceed to τ.

Definition 6 [32] Let three BCFVs $\tau =\left(\delta \left(x\right)+i\theta \left(x\right),\nu \left(x\right)+i\varphi \left(x\right)\right),\tau_{1}=\left(\delta_{1}\left(x\right)+i\theta_{1}\left(x\right),\nu_{1}\left(x\right)+i\varphi_{1}\left(x\right)\right),\tau_{2}=\left(\delta_{2}\left(x\right)+i\theta_{2}\left(x\right),\nu_{2}\left(x\right)+i\varphi_{2}\left(x\right)\right)$ and λ>0. Then, some robust rules are defined as follows:

a $\tau_{1}⊕\tau_{1}=\left(\begin{array}{c} \delta_{1}\left(x\right)+\delta_{2}\left(x\right)-\delta_{1}\left(x\right)\delta_{2}\left(x\right)+i\left(\theta_{1}\left(x\right)+\theta_{2}\left(x\right)-\theta_{1}\left(x\right)\theta_{2}\left(x\right)\right),\\ -\left(\nu_{1}\left(x\right)\nu_{2}\left(x\right)\right)+i\left(-\left(\varphi_{1}\left(x\right)\varphi_{2}\left(x\right)\right)\right) \end{array}\right)$
b $\tau_{1}⊗\tau_{1}=\left(\begin{array}{c} \delta_{1}\left(x\right)\delta_{2}\left(x\right)+i\theta_{1}\left(x\right)\theta_{2}\left(x\right)\\ \nu_{1}\left(x\right)+\nu_{2}\left(x\right)+\nu_{1}\left(x\right)\nu_{2}\left(x\right)+i\left(\varphi_{1}\left(x\right)+\varphi_{2}\left(x\right)+\varphi_{1}\left(x\right)\varphi_{2}\left(x\right)\right) \end{array}\right)$
c $\lambda \tau =\left(\begin{array}{c} 1-{\left(1-\delta \left(x\right)\right)}^{\lambda }+i\left(1-{\left(1-\theta \left(x\right)\right)}^{\lambda }\right),\\ -{\left|\nu \left(x\right)\right|}^{\lambda }+i\left(-{\left|\varphi \left(x\right)\right|}^{\lambda }\right) \end{array}\right)$
d $\tau^{\lambda }=\left(\begin{array}{c} \delta^{\lambda }\left(x\right)+i\theta^{\lambda }\left(x\right),\\ -1+{\left(1+\nu \left(x\right)\right)}^{\lambda }+i\left(-1+{\left(1+\varphi \left(x\right)\right)}^{\lambda }\right) \end{array}\right)$

Theorem 1 [32] *Let three BCFVs* $\tau =\left(\delta \left(x\right)+i\theta \left(x\right),\nu \left(x\right)+i\varphi \left(x\right)\right),\tau_{1}=\left(\delta_{1}\left(x\right)+i\theta_{1}\left(x\right),\nu_{1}\left(x\right)+i\varphi_{1}\left(x\right)\right)$ *and*$\tau_{2}=\left(\delta_{2}\left(x\right)+i\theta_{2}\left(x\right),\nu_{2}\left(x\right)+i\varphi_{2}\left(x\right)\right)$ *with real number* β,β1,β2>0. *Then*:

a $\tau_{1}⊕\tau_{2}=\tau_{1}⊕\tau_{2}$
b $\tau_{1}⊗\tau_{2}=\tau_{1}⊗\tau_{2}$
c $\beta \left(\tau_{1}⊕\tau_{2}\right)=\beta \tau_{1}⊕\beta \tau_{2}$
d ${\left(\tau_{1}⊗\tau_{2}\right)}^{\beta }={\tau_{1}}^{\beta }⊗\tau_{2}^{\beta }$
e $\beta_{1}\tau ⊕\beta_{2}\tau =\left(\beta_{1}+\beta_{2}\right)\tau$
f $\tau_{1}^{\beta_{1}}⊗\tau_{1}^{\beta_{2}}=\tau^{\beta_{1}+\beta_{2}}$
g ${\left(\tau^{\beta_{1}}\right)}^{\beta_{2}}=\tau^{\beta_{1}\beta_{2}}$
h $\tau_{1}⊕\left(\tau_{2}⊕\tau \right)=\left(\tau_{1}⊕\tau_{2}\right)⊕\tau$
i $\tau_{1}⊗\left(\tau_{2}⊗\tau \right)=\left(\tau_{1}⊗\tau_{1}\right)⊗\tau$
j $\beta_{1}\left(\beta_{2}\tau_{1}\right)=\left(\beta_{1}\beta_{2}\right)\tau_{1}$

Definition 7 [56] *Consider* $\varphi_{i},i\ldots ‥,n$ *be a class of positive integers*. *The Hamy mean* (*HM*) *operator is expressed in the following* Eq. (3) *and we have*:

(3) $${HM}^{\left(\partial \right)}\left(\varphi_{1},\varphi_{2}.\;.\;.,\varphi_{n}\right)=\frac{\sum_{\;1\leq i_{1}<\ldots \;{<i}_{\partial }\leq n}{\left(\prod_{j=1}^{\partial }\varphi_{ij}\right)}^{\frac{1}{\partial }}}{C_{n}^{\partial }}$$

Where ∂ is a parameter, $\partial =1,2,.\;.\;.,n,i_{1},i_{2},.\;.\;.,i_{\partial }$ are integer values taken from the set {1,2,....,n} of k integer values and $C_{n}^{\partial }$ is the binomial coefficient. i.e $C_{n}^{\partial }=\frac{n!}{\partial !\left(n-\partial \right)!}$.

Definition 8 [56] Consider $\varphi_{i},i\ldots ‥,n$ be a class of positive integers. The Dual Hamy mean (DHM) operator is expressed in Eq. (4) and can be written as:

(4) $$DHM^{\left(\partial \right)}\left(\varphi_{1},\varphi_{2}.\;.\;.,\varphi_{n}\right)={\left(\prod_{1\leq i_{1}<\ldots \;{<i}_{\partial }\leq n}\left(\frac{\sum_{j=1}^{\partial }\varphi_{ij}}{\partial }\right)\right)}^{\frac{1}{C_{n}^{\partial }}}$$

## 3 Bipolar complex fuzzy Hamy mean aggregation operators

Now, we derive a family of new mathematical approaches with Hamy mean operators under consideration in the system of bipolar complex fuzzy environments.

Definition 9 Let BCFVs $\tau_{j}=\left(\delta_{j}\left(x\right)+i\theta_{j}\left(x\right),\nu_{j}\left(x\right)+i\varphi_{j}\left(x\right)\right),j=1,2,\ldots ,p$. The BCFHM operator is particularized in Eq. (5) and written as follows:

(5) $${BCFHM}^{\left(\partial \right)}\left(\tau_{1},\tau_{2}.\;.\;.,\tau_{p}\right)=\frac{\underset{1\leq {ℷ}_{1}<\ldots \;{<ℷ}_{\partial }\leq p}{⨁}{\left(\overset{\partial }{\underset{ℷ=1}{⨂}}\tau_{j_{ℷ}}\right)}^{\frac{1}{\partial }}}{C_{p}^{\partial }}$$

Theorem 2 *Let BCFVs*$\tau_{j}=\left(\delta_{j}\left(x\right)+i\theta_{j}\left(x\right),\nu_{j}\left(x\right)+i\varphi_{j}\left(x\right)\right),j=1,2,\ldots ,p$. *Then*, *the aggregated outcome by the BCFHM operator is also a BCFV and the above expression can be written as* Eq. (6):

(6) $${BCFHM}^{\left(\partial \right)}\left(\tau_{1},\tau_{2}.\;.\;.,\tau_{p}\right)=\left(\begin{array}{c} 1-{\left(\prod_{1\leq {ℷ}_{1}<\ldots \;{<ℷ}_{\partial }\leq p}\left(1-{\left(\prod_{ℷ=1}^{\partial }\delta_{j_{ℷ}}\left(x\right)\right)}^{\frac{1}{\partial }}\right)\right)}^{\frac{1}{C_{p}^{\partial }}}+\\ i\left(1-{\left(\prod_{1\leq {ℷ}_{1}<\ldots \;{<ℷ}_{\partial }\leq p}\left(1-{\left(\prod_{ℷ=1}^{\partial }\theta_{j_{ℷ}}\left(x\right)\right)}^{\frac{1}{\partial }}\right)\right)}^{\frac{1}{C_{p}^{\partial }}}\right),\\ -{\left|-\left(\prod_{1\leq {ℷ}_{1}<\ldots \;{<ℷ}_{\partial }\leq p}\left(-1+{\left(\prod_{ℷ=1}^{\partial }\left(1+\nu_{j_{ℷ}}\left(x\right)\right)\right)}^{\frac{1}{\partial }}\right)\right)\right|}^{\frac{1}{C_{p}^{\partial }}}+\\ i\left(-{\left|-\left(\prod_{1\leq {ℷ}_{1}<\ldots \;{<ℷ}_{\partial }\leq p}\left(-1+{\left(\prod_{ℷ=1}^{\partial }\left(1+\varphi_{j_{ℷ}}\left(x\right)\right)\right)}^{\frac{1}{\partial }}\right)\right)\right|}^{\frac{1}{C_{p}^{\partial }}}\right) \end{array}\right)$$

Proof *We know that a family of BCFVs*$\tau_{j}=\left(\delta_{j}\left(x\right)+i\theta_{j}\left(x\right),\nu_{j}\left(x\right)+i\varphi_{j}\left(x\right)\right),j=1,2,\ldots ,p$. *We can prove the above expression under the following steps*:

$$\overset{\partial }{\underset{ℷ=1}{⨂}}\tau_{j_{ℷ}}=\left(\begin{array}{c} \prod_{ℷ=1}^{\partial }\delta_{j_{ℷ}}\left(x\right)+i\prod_{ℷ=1}^{\partial }\theta_{j_{ℷ}}\left(x\right)\\ -1+\prod_{ℷ=1}^{\partial }\left(1+\nu_{j_{ℷ}}\left(x\right)\right)+i\left(-1+\prod_{ℷ=1}^{\partial }\left(1+\varphi_{j_{ℷ}}\left(x\right)\right)\right) \end{array}\right)$$

$${\left(\overset{\partial }{\underset{ℷ=1}{⨂}}\tau_{j_{ℷ}}\right)}^{\frac{1}{\partial }}=\left(\begin{array}{c} {\left(\prod_{ℷ=1}^{\partial }\delta_{j_{ℷ}}\left(x\right)\right)}^{\frac{1}{\partial }}+i{\left(\prod_{ℷ=1}^{\partial }\theta_{j_{ℷ}}\left(x\right)\right)}^{\frac{1}{\partial }},\\ -1+{\left(\prod_{ℷ=1}^{\partial }\left(1+\nu_{j_{ℷ}}\left(x\right)\right)\right)}^{\frac{1}{\partial }}+i\left(-1+{\left(\prod_{ℷ=1}^{\partial }\left(1+\varphi_{j_{ℷ}}\left(x\right)\right)\right)}^{\frac{1}{\partial }}\right) \end{array}\right)$$

$$\underset{1\leq {ℷ}_{1}<\ldots \;{<ℷ}_{\partial }\leq p}{⨁}{\left(\overset{\partial }{\underset{ℷ=1}{⨂}}\tau_{j_{ℷ}}\right)}^{\frac{1}{\partial }}=\left(\begin{array}{c} 1-\prod_{1\leq {ℷ}_{1}<\ldots \;{<ℷ}_{\partial }\leq p}\left(1-{\left(\prod_{ℷ=1}^{\partial }\delta_{j_{ℷ}}\left(x\right)\right)}^{\frac{1}{\partial }}\right)+\\ i\left(1-\prod_{1\leq {ℷ}_{1}<\ldots \;{<ℷ}_{\partial }\leq p}\left(1-{\left(\prod_{ℷ=1}^{\partial }\theta_{j_{ℷ}}\left(x\right)\right)}^{\frac{1}{\partial }}\right)\right),\\ -\left(\prod_{1\leq {ℷ}_{1}<\ldots \;{<ℷ}_{\partial }\leq p}\left(-1+{\left(\prod_{ℷ=1}^{\partial }\left(1+\nu_{j_{ℷ}}\left(x\right)\right)\right)}^{\frac{1}{\partial }}\right)\right)+\\ i\left(-\left(\prod_{1\leq {ℷ}_{1}<\ldots \;{<ℷ}_{\partial }\leq p}\left(-1+{\left(\prod_{ℷ=1}^{\partial }\left(1+\varphi_{j_{ℷ}}\left(x\right)\right)\right)}^{\frac{1}{\partial }}\right)\right)\right) \end{array}\right)$$

$$\frac{\underset{1\leq {ℷ}_{1}<\ldots \;{<ℷ}_{\partial }\leq p}{⨁}{\left(\overset{\partial }{\underset{ℷ=1}{⨂}}\tau_{j_{ℷ}}\right)}^{\frac{1}{\partial }}}{C_{p}^{\partial }}=\left(\begin{array}{c} 1-{\left(\prod_{1\leq {ℷ}_{1}<\ldots \;{<ℷ}_{\partial }\leq p}\left(1-{\left(\prod_{ℷ=1}^{\partial }\delta_{j_{ℷ}}\left(x\right)\right)}^{\frac{1}{\partial }}\right)\right)}^{\frac{1}{C_{p}^{\partial }}}+\\ i\left(1-{\left(\prod_{1\leq {ℷ}_{1}<\ldots \;{<ℷ}_{\partial }\leq p}\left(1-{\left(\prod_{ℷ=1}^{\partial }\theta_{j_{ℷ}}\left(x\right)\right)}^{\frac{1}{\partial }}\right)\right)}^{\frac{1}{C_{p}^{\partial }}}\right),\\ -{\left|-\left(\prod_{1\leq {ℷ}_{1}<\ldots \;{<ℷ}_{\partial }\leq p}\left(-1+{\left(\prod_{ℷ=1}^{\partial }\left(1+\nu_{j_{ℷ}}\left(x\right)\right)\right)}^{\frac{1}{\partial }}\right)\right)\right|}^{\frac{1}{C_{p}^{\partial }}}+\\ i\left(-{\left|-\left(\prod_{1\leq {ℷ}_{1}<\ldots \;{<ℷ}_{\partial }\leq p}\left(-1+{\left(\prod_{ℷ=1}^{\partial }\left(1+\varphi_{j_{ℷ}}\left(x\right)\right)\right)}^{\frac{1}{\partial }}\right)\right)\right|}^{\frac{1}{C_{p}^{\partial }}}\right) \end{array}\right)$$

Property 1 *Let a family of BCFVs*$\tau_{j}=\left(\delta_{j}\left(x\right)+i\theta_{j}\left(x\right),\nu_{j}\left(x\right)+i\varphi_{j}\left(x\right)\right),j=1,2,\ldots ,n$*such that*$\tau_{j}=\tau$. *Then*:

$$BCFHM\left(\tau_{1},\tau_{2},\ldots ,\tau_{n}\right)=\tau$$

Property 2 *Suppose that two sets of BCFVs*$\tau_{j}=\left(\delta_{j}\left(x\right)+i\theta_{j}\left(x\right),\nu_{j}\left(x\right)+i\varphi_{j}\left(x\right)\right)$*and*$\tau_{j}^{\prime }=\left(\delta_{j}^{\prime }\left(x\right)+i\theta_{j}^{\prime }\left(x\right),\nu_{j}^{\prime }\left(x\right)+i\varphi_{j}^{\prime }\left(x\right)\right),j=1,2,\ldots ,n$. *If*$\tau_{j}\leq \tau_{j}^{\prime }$*such that*$\delta_{j}\left(x\right)\leq \delta_{j}^{\prime }\left(x\right),\theta_{j}\left(x\right)\leq \theta_{j}^{\prime }\left(x\right)$*and*$\nu_{j}\left(x\right)\geq \nu_{j}^{\prime }\left(x\right),\varphi_{j}\left(x\right)\geq \varphi_{j}^{\prime }\left(x\right)$. *Then*:

$$BCFHM\left(\tau_{1},\tau_{2},\ldots ,\tau_{n}\right)\leq BCFHM\left(\tau_{1}^{\prime },\tau_{2}^{\prime },\ldots ,\tau_{n}^{\prime }\right)$$

Property 3 *Let a family of BCFVs*$\tau_{j}=\left(\delta_{j}\left(x\right)+i\theta_{j}\left(x\right),\nu_{j}\left(x\right)+i\varphi_{j}\left(x\right)\right),j=1,2,\ldots ,n$. *If*$\tau^{-}=\min\left(\tau_{1},\tau_{2},\ldots ,\tau_{n}\right)$*and*$\tau^{+}=\max\left(\tau_{1},\tau_{2},\ldots ,\tau_{n}\right)$. *Then*:

$$\tau^{-}\leq BCFHM\left(\tau_{1},\tau_{2},\ldots ,\tau_{n}\right)\leq \tau^{+}$$

Definition 10 Let a family of BCFVs $\tau_{j}=\left(\delta_{j}\left(x\right)+i\theta_{j}\left(x\right),\nu_{j}\left(x\right)+i\varphi_{j}\left(x\right)\right),j=1,2,\ldots ,n$ with weight vector $w={\left(w_{1},w_{2},\ldots ,w_{n}\right)}^{T},w_{i}\in \left[0,1\right]$ and $\sum_{i=1}^{n}w_{i}=1$. The BCFWHM operator is expressed in the following Eq. (7), and we have:

(7) $${BCFWHM}^{\left(\partial \right)}\left(\tau_{1},\tau_{2}.\;.\;.,\tau_{p}\right)=\left\{ \begin{array}{cc} \frac{\underset{1\leq {ℷ}_{1}<\ldots \;{<ℷ}_{\partial }\leq p}{⨁}\left(1-\sum_{ℷ=1}^{\partial }w_{i_{j}}\right){\left(\overset{\partial }{\underset{ℷ=1}{⨂}}\tau_{j_{ℷ}}\right)}^{\frac{1}{\partial }}}{C_{p-1}^{\partial }} & 1\leq \partial <p\\ \overset{\partial }{\underset{ℷ=1}{⨂}}\tau_{j_{ℷ}}^{\frac{1-w_{i}}{p-1}} & \partial =p \end{array}\right.$$

Theorem 3 *Let a family of BCFVs*$\tau_{j}=\left(\delta_{j}\left(x\right)+i\theta_{j}\left(x\right),\nu_{j}\left(x\right)+i\varphi_{j}\left(x\right)\right),j=1,2,\ldots ,n$*with weight vector*$w={\left(w_{1},w_{2},\ldots ,w_{n}\right)}^{T},w_{i}\in \left[0,1\right]$*and*$\sum_{i=1}^{n}w_{i}=1$. *Then*, *the aggregated outcome of the BCFWHM operator is also a BCFV*; *the above expression can be written as the following* Eq. (8):

(8) $${BCFWHM}^{\left(\partial \right)}\left(\tau_{1},\tau_{2}.\;.\;.,\tau_{p}\right)=\left(\begin{array}{c} 1-{\left(\prod_{1\leq {ℷ}_{1}<\ldots \;{<ℷ}_{\partial }\leq p}\left({\left(1-{\left(\prod_{ℷ=1}^{\partial }\delta_{j_{ℷ}}\left(x\right)\right)}^{\frac{1}{\partial }}\right)}^{\left(1-\sum_{ℷ=1}^{\partial }w_{i_{j}}\right)}\right)\right)}^{\frac{1}{C_{p-1}^{\partial }}}+\\ i\left(1-{\left(\prod_{1\leq {ℷ}_{1}<\ldots \;{<ℷ}_{\partial }\leq p}\left({\left(1-{\left(\prod_{ℷ=1}^{\partial }\theta_{j_{ℷ}}\left(x\right)\right)}^{\frac{1}{\partial }}\right)}^{\left(1-\sum_{ℷ=1}^{\partial }w_{i_{j}}\right)}\right)\right)}^{\frac{1}{C_{p-1}^{\partial }}}\right),\\ -{\left|-\left(\prod_{1\leq {ℷ}_{1}<\ldots \;{<ℷ}_{\partial }\leq p}-{\left|-1+{\left(\prod_{ℷ=1}^{\partial }\left(1+\nu_{j_{ℷ}}\left(x\right)\right)\right)}^{\frac{1}{\partial }}\right|}^{\left(1-\sum_{ℷ=1}^{\partial }w_{i_{j}}\right)}\right)\right|}^{\frac{1}{C_{p-1}^{\partial }}}+\\ i\left(-{\left|-\left(\prod_{1\leq {ℷ}_{1}<\ldots \;{<ℷ}_{\partial }\leq p}-{\left|-1+{\left(\prod_{ℷ=1}^{\partial }\left(1+\varphi_{j_{ℷ}}\left(x\right)\right)\right)}^{\frac{1}{\partial }}\right|}^{\left(1-\sum_{ℷ=1}^{\partial }w_{i_{j}}\right)}\right)\right|}^{\frac{1}{C_{p-1}^{\partial }}}\right) \end{array}\right),1\leq \partial <p=\left(\begin{array}{c} \prod_{ℷ=1}^{\partial }{\left(\delta_{j_{ℷ}}\left(x\right)\right)}^{\left(\frac{1-w_{i}}{p-1}\right)}+\\ i\prod_{ℷ=1}^{\partial }{\left(\theta_{j_{ℷ}}\left(x\right)\right)}^{\left(\frac{1-w_{i}}{p-1}\right)}\\ -1+\prod_{ℷ=1}^{\partial }\left({\left(1+\nu_{j_{ℷ}}\left(x\right)\right)}^{\left(\frac{1-w_{i}}{p-1}\right)}\right)+\\ i\left(-1+\prod_{ℷ=1}^{\partial }\left({\left(1+\varphi_{j_{ℷ}}\left(x\right)\right)}^{\left(\frac{1-w_{i}}{p-1}\right)}\right)\right) \end{array}\right),\partial =p$$

Proof Since a family of BCFVs $\tau_{j}=\left(\delta_{j}\left(x\right)+i\theta_{j}\left(x\right),\nu_{j}\left(x\right)+i\varphi_{j}\left(x\right)\right),j=1,2,\ldots ,n$ and above expressions are verified under the following steps:

Case 1 Suppose that for 1≤∂<p.

$$\overset{\partial }{\underset{ℷ=1}{⨂}}\tau_{j_{ℷ}}=\left(\begin{array}{c} \prod_{ℷ=1}^{\partial }\delta_{j_{ℷ}}\left(x\right)+i\prod_{ℷ=1}^{\partial }\theta_{j_{ℷ}}\left(x\right)\\ -1+\prod_{ℷ=1}^{\partial }\left(1+\nu_{j_{ℷ}}\left(x\right)\right)+i\left(-1+\prod_{ℷ=1}^{\partial }\left(1+\varphi_{j_{ℷ}}\left(x\right)\right)\right) \end{array}\right)$$

$${\left(\overset{\partial }{\underset{ℷ=1}{⨂}}\tau_{j_{ℷ}}\right)}^{\frac{1}{\partial }}=\left(\begin{array}{c} {\left(\prod_{ℷ=1}^{\partial }\delta_{j_{ℷ}}\left(x\right)\right)}^{\frac{1}{\partial }}+i{\left(\prod_{ℷ=1}^{\partial }\theta_{j_{ℷ}}\left(x\right)\right)}^{\frac{1}{\partial }},\\ -1+{\left(\prod_{ℷ=1}^{\partial }\left(1+\nu_{j_{ℷ}}\left(x\right)\right)\right)}^{\frac{1}{\partial }}+i\left(-1+{\left(\prod_{ℷ=1}^{\partial }\left(1+\varphi_{j_{ℷ}}\left(x\right)\right)\right)}^{\frac{1}{\partial }}\right) \end{array}\right)$$

$$\left(1-\sum_{ℷ=1}^{\partial }w_{i_{j}}\right){\left(\overset{\partial }{\underset{ℷ=1}{⨂}}\tau_{j_{ℷ}}\right)}^{\frac{1}{\partial }}=\left(\begin{array}{c} 1-{\left(1-{\left(\prod_{ℷ=1}^{\partial }\delta_{j_{ℷ}}\left(x\right)\right)}^{\frac{1}{\partial }}\right)}^{\left(1-\sum_{ℷ=1}^{\partial }w_{i_{j}}\right)}+i\left(1-{\left(1-{\left(\prod_{ℷ=1}^{\partial }\theta_{j_{ℷ}}\left(x\right)\right)}^{\frac{1}{\partial }}\right)}^{\left(1-\sum_{ℷ=1}^{\partial }w_{i_{j}}\right)}\right),\\ -{\left|-1+{\left(\prod_{ℷ=1}^{\partial }\left(1+\nu_{j_{ℷ}}\left(x\right)\right)\right)}^{\frac{1}{\partial }}\right|}^{\left(1-\sum_{ℷ=1}^{\partial }w_{i_{j}}\right)}+i\left(-{\left|-1+{\left(\prod_{ℷ=1}^{\partial }\left(1+\varphi_{j_{ℷ}}\left(x\right)\right)\right)}^{\frac{1}{\partial }}\right|}^{\left(1-\sum_{ℷ=1}^{\partial }w_{i_{j}}\right)}\right) \end{array}\right)$$

$$\underset{1\leq {ℷ}_{1}<\ldots \;{<ℷ}_{\partial }\leq p}{⨁}\left(1-\sum_{ℷ=1}^{\partial }w_{i_{j}}\right){\left(\overset{\partial }{\underset{ℷ=1}{⨂}}\tau_{j_{ℷ}}\right)}^{\frac{1}{\partial }}=\left(\begin{array}{c} 1-\prod_{1\leq {ℷ}_{1}<\ldots \;{<ℷ}_{\partial }\leq p}\left({\left(1-{\left(\prod_{ℷ=1}^{\partial }\delta_{j_{ℷ}}\left(x\right)\right)}^{\frac{1}{\partial }}\right)}^{\left(1-\sum_{ℷ=1}^{\partial }w_{i_{j}}\right)}\right)+\\ i\left(1-\prod_{1\leq {ℷ}_{1}<\ldots \;{<ℷ}_{\partial }\leq p}\left({\left(1-{\left(\prod_{ℷ=1}^{\partial }\theta_{j_{ℷ}}\left(x\right)\right)}^{\frac{1}{\partial }}\right)}^{\left(1-\sum_{ℷ=1}^{\partial }w_{i_{j}}\right)}\right)\right),\\ -\left(\prod_{1\leq {ℷ}_{1}<\ldots \;{<ℷ}_{\partial }\leq p}-{\left|-1+{\left(\prod_{ℷ=1}^{\partial }\left(1+\nu_{j_{ℷ}}\left(x\right)\right)\right)}^{\frac{1}{\partial }}\right|}^{\left(1-\sum_{ℷ=1}^{\partial }w_{i_{j}}\right)}\right)+\\ i\left(-\left(\prod_{1\leq {ℷ}_{1}<\ldots \;{<ℷ}_{\partial }\leq p}-{\left|-1+{\left(\prod_{ℷ=1}^{\partial }\left(1+\varphi_{j_{ℷ}}\left(x\right)\right)\right)}^{\frac{1}{\partial }}\right|}^{\left(1-\sum_{ℷ=1}^{\partial }w_{i_{j}}\right)}\right)\right) \end{array}\right)$$

$$\frac{\underset{1\leq {ℷ}_{1}<\ldots \;{<ℷ}_{\partial }\leq p}{⨁}\left(1-\sum_{ℷ=1}^{\partial }w_{i_{j}}\right){\left(\overset{\partial }{\underset{ℷ=1}{⨂}}\tau_{j_{ℷ}}\right)}^{\frac{1}{\partial }}}{C_{p-1}^{\partial }}=\left(\begin{array}{c} 1-{\left(\prod_{1\leq {ℷ}_{1}<\ldots \;{<ℷ}_{\partial }\leq p}\left({\left(1-{\left(\prod_{ℷ=1}^{\partial }\delta_{j_{ℷ}}\left(x\right)\right)}^{\frac{1}{\partial }}\right)}^{\left(1-\sum_{ℷ=1}^{\partial }w_{i_{j}}\right)}\right)\right)}^{\frac{1}{C_{p-1}^{\partial }}}+\\ i\left(1-{\left(\prod_{1\leq {ℷ}_{1}<\ldots \;{<ℷ}_{\partial }\leq p}\left({\left(1-{\left(\prod_{ℷ=1}^{\partial }\theta_{j_{ℷ}}\left(x\right)\right)}^{\frac{1}{\partial }}\right)}^{\left(1-\sum_{ℷ=1}^{\partial }w_{i_{j}}\right)}\right)\right)}^{\frac{1}{C_{p-1}^{\partial }}}\right),\\ -{\left|-\left(\prod_{1\leq {ℷ}_{1}<\ldots \;{<ℷ}_{\partial }\leq p}-{\left|-1+{\left(\prod_{ℷ=1}^{\partial }\left(1+\nu_{j_{ℷ}}\left(x\right)\right)\right)}^{\frac{1}{\partial }}\right|}^{\left(1-\sum_{ℷ=1}^{\partial }w_{i_{j}}\right)}\right)\right|}^{\frac{1}{C_{p-1}^{\partial }}}+\\ i\left(-{\left|-\left(\prod_{1\leq {ℷ}_{1}<\ldots \;{<ℷ}_{\partial }\leq p}-{\left|-1+{\left(\prod_{ℷ=1}^{\partial }\left(1+\varphi_{j_{ℷ}}\left(x\right)\right)\right)}^{\frac{1}{\partial }}\right|}^{\left(1-\sum_{ℷ=1}^{\partial }w_{i_{j}}\right)}\right)\right|}^{\frac{1}{C_{p-1}^{\partial }}}\right) \end{array}\right)$$

Case 2 Suppose that for ∂=p.

$$\tau_{j_{ℷ}}^{\frac{1-w_{i}}{p-1}}=\left(\begin{array}{c} {\left(\delta_{j_{ℷ}}\left(x\right)\right)}^{\left(\frac{1-w_{i}}{p-1}\right)}+i{\left(\theta_{j_{ℷ}}\left(x\right)\right)}^{\left(\frac{1-w_{i}}{p-1}\right)},\\ -1+{\left(1+\nu_{j_{ℷ}}\left(x\right)\right)}^{\left(\frac{1-w_{i}}{p-1}\right)}+i\left(-1+{\left(1+\varphi_{j_{ℷ}}\left(x\right)\right)}^{\left(\frac{1-w_{i}}{p-1}\right)}\right) \end{array}\right)$$

$$\overset{\partial }{\underset{ℷ=1}{⨂}}\tau_{j_{ℷ}}^{\frac{1-w_{i}}{p-1}}=\left(\begin{array}{c} \prod_{ℷ=1}^{\partial }{\left(\delta_{j_{ℷ}}\left(x\right)\right)}^{\left(\frac{1-w_{i}}{p-1}\right)}+\\ i\prod_{ℷ=1}^{\partial }{\left(\theta_{j_{ℷ}}\left(x\right)\right)}^{\left(\frac{1-w_{i}}{p-1}\right)}\\ -1+\prod_{ℷ=1}^{\partial }\left({\left(1+\nu_{j_{ℷ}}\left(x\right)\right)}^{\left(\frac{1-w_{i}}{p-1}\right)}\right)+\\ i\left(-1+\prod_{ℷ=1}^{\partial }\left({\left(1+\varphi_{j_{ℷ}}\left(x\right)\right)}^{\left(\frac{1-w_{i}}{p-1}\right)}\right)\right) \end{array}\right)$$

Property 4 *Let a family of BCFVs*$\tau_{j}=\left(\delta_{j}\left(x\right)+i\theta_{j}\left(x\right),\nu_{j}\left(x\right)+i\varphi_{j}\left(x\right)\right),j=1,2,\ldots ,n$*such that*$\tau_{j}=\tau$. *Then*:

$$BCFWHM\left(\tau_{1},\tau_{2},\ldots ,\tau_{n}\right)=\tau$$

Property 5 *Suppose that two sets of BCFVs*$\tau_{j}=\left(\delta_{j}\left(x\right)+i\theta_{j}\left(x\right),\nu_{j}\left(x\right)+i\varphi_{j}\left(x\right)\right)$*and*$\tau_{j}^{\prime }=\left(\delta_{j}^{\prime }\left(x\right)+i\theta_{j}^{\prime }\left(x\right),\nu_{j}^{\prime }\left(x\right)+i\varphi_{j}^{\prime }\left(x\right)\right),j=1,2,\ldots ,n$. *If*$\tau_{j}\leq \tau_{j}^{\prime }$*such that*$\delta_{j}\left(x\right)\leq \delta_{j}^{\prime }\left(x\right),\theta_{j}\left(x\right)\leq \theta_{j}^{\prime }\left(x\right)$*and*$\nu_{j}\left(x\right)\geq \nu_{j}^{\prime }\left(x\right),\varphi_{j}\left(x\right)\geq \varphi_{j}^{\prime }\left(x\right)$. *Then*:

$$BCFWHM\left(\tau_{1},\tau_{2},\ldots ,\tau_{n}\right)\leq BCFWHM\left(\tau_{1}^{\prime },\tau_{2}^{\prime },\ldots ,\tau_{n}^{\prime }\right)$$

Property 6 *Let a family of BCFVs*$\tau_{j}=\left(\delta_{j}\left(x\right)+i\theta_{j}\left(x\right),\nu_{j}\left(x\right)+i\varphi_{j}\left(x\right)\right),j=1,2,\ldots ,n$. *If*$\tau^{-}=\min\left(\tau_{1},\tau_{2},\ldots ,\tau_{n}\right)$*and*$\tau^{+}=\max\left(\tau_{1},\tau_{2},\ldots ,\tau_{n}\right)$. *Then*:

$$\tau^{-}\leq BCFWHM\left(\tau_{1},\tau_{2},\ldots ,\tau_{n}\right)\leq \tau^{+}$$

## 4 Bipolar complex fuzzy Dual Hamy mean aggregation operators

This section presents a list of dominant mathematical approaches and strategies by generalizing the theory of DHM operators based on BCF information.

Definition 11 Let BCFVs $\tau_{j}=\left(\delta_{j}+i\theta_{j},\nu_{j}+i\varphi_{j}\right),j=1,2,\ldots ,p$. The BCFDHM operator is particularized in the following Eq. (9):

(9) $${BCFDHM}^{\left(\partial \right)}\left(\tau_{1},\tau_{2}.\;.\;.,\tau_{p}\right)={\left(\underset{1\leq {ℷ}_{1}<\ldots \;{<ℷ}_{\partial }\leq p}{⨂}\frac{\left(\overset{\partial }{\underset{ℷ=1}{⨁}}\tau_{j_{ℷ}}\right)}{\partial }\right)}^{\frac{1}{C_{p-1}^{\partial }}}$$

Theorem 4 *Let BCFVs*$\tau_{j}=\left(\delta_{j}+i\theta_{j},\nu_{j}+i\varphi_{j}\right),j=1,2,\ldots ,p$. *Then*, *the aggregated outcome by the BCFHM operator is also a BCFV*; *the above expression can be written as following* Eq. (10):

(10) $${BCFDHM}^{\left(\partial \right)}\left(\tau_{1},\tau_{2}.\;.\;.,\tau_{p}\right)=\left(\begin{array}{c} {\left(\prod_{1\leq {ℷ}_{1}<\ldots \;{<ℷ}_{\partial }\leq p}\left(1-{\left(\prod_{ℷ=1}^{\partial }\left(1-\delta_{j_{ℷ}}\left(x\right)\right)\right)}^{\frac{1}{\partial }}\right)\right)}^{\frac{1}{C_{p-1}^{\partial }}}+\\ i{\left(\prod_{1\leq {ℷ}_{1}<\ldots \;{<ℷ}_{\partial }\leq p}\left(1-{\left(\prod_{ℷ=1}^{\partial }\left(1-\theta_{j_{ℷ}}\left(x\right)\right)\right)}^{\frac{1}{\partial }}\right)\right)}^{\frac{1}{C_{p-1}^{\partial }}},\\ -1+{\left(\prod_{1\leq {ℷ}_{1}<\ldots \;{<ℷ}_{\partial }\leq p}\left(1-{\left|-\left(\prod_{ℷ=1}^{\partial }\nu_{j_{ℷ}}\left(x\right)\right)\right|}^{\frac{1}{\partial }}\right)\right)}^{\frac{1}{C_{p-1}^{\partial }}}+\\ i\left(-1+{\left(\prod_{1\leq {ℷ}_{1}<\ldots \;{<ℷ}_{\partial }\leq p}\left(1-{\left|-\left(\prod_{ℷ=1}^{\partial }\varphi_{j_{ℷ}}\left(x\right)\right)\right|}^{\frac{1}{\partial }}\right)\right)}^{\frac{1}{C_{p-1}^{\partial }}}\right) \end{array}\right)$$

Proof To prove the above expression, we use the traditional operational laws discussed in Definition 7 as follows:

$$\left(\overset{\partial }{\underset{ℷ=1}{⨁}}\tau_{j_{ℷ}}\right)=\left(\begin{array}{c} 1-\prod_{ℷ=1}^{\partial }\left(1-\delta_{j_{ℷ}}\left(x\right)\right)+i\left(1-\prod_{ℷ=1}^{\partial }\left(1-\theta_{j_{ℷ}}\left(x\right)\right)\right),\\ -\left(\prod_{ℷ=1}^{\partial }\nu_{j_{ℷ}}\left(x\right)\right)+i\left(-\left(\prod_{ℷ=1}^{\partial }\varphi_{j_{ℷ}}\left(x\right)\right)\right) \end{array}\right)$$

$$\frac{\left(\overset{\partial }{\underset{ℷ=1}{⨁}}\tau_{j_{ℷ}}\right)}{\partial }=\left(\begin{array}{c} 1-{\left(\prod_{ℷ=1}^{\partial }\left(1-\delta_{j_{ℷ}}\left(x\right)\right)\right)}^{\frac{1}{\partial }}+i\left(1-{\left(\prod_{ℷ=1}^{\partial }\left(1-\theta_{j_{ℷ}}\left(x\right)\right)\right)}^{\frac{1}{\partial }}\right),\\ -{\left|-\left(\prod_{ℷ=1}^{\partial }\nu_{j_{ℷ}}\left(x\right)\right)\right|}^{\frac{1}{\partial }}+i\left(-{\left|-\left(\prod_{ℷ=1}^{\partial }\varphi_{j_{ℷ}}\left(x\right)\right)\right|}^{\frac{1}{\partial }}\right) \end{array}\right)$$

$$\underset{1\leq {ℷ}_{1}<\ldots \;{<ℷ}_{\partial }\leq p}{⨂}\frac{\left(\overset{\partial }{\underset{ℷ=1}{⨁}}\tau_{j_{ℷ}}\right)}{\partial }=\left(\begin{array}{c} \prod_{1\leq {ℷ}_{1}<\ldots \;{<ℷ}_{\partial }\leq p}\left(1-{\left(\prod_{ℷ=1}^{\partial }\left(1-\delta_{j_{ℷ}}\left(x\right)\right)\right)}^{\frac{1}{\partial }}\right)+\\ i\prod_{1\leq {ℷ}_{1}<\ldots \;{<ℷ}_{\partial }\leq p}\left(1-{\left(\prod_{ℷ=1}^{\partial }\left(1-\theta_{j_{ℷ}}\left(x\right)\right)\right)}^{\frac{1}{\partial }}\right)\\ -1+\prod_{1\leq {ℷ}_{1}<\ldots \;{<ℷ}_{\partial }\leq p}\left(1-{\left|-\left(\prod_{ℷ=1}^{\partial }\nu_{j_{ℷ}}\left(x\right)\right)\right|}^{\frac{1}{\partial }}\right)+\\ i\left(-1+\prod_{1\leq {ℷ}_{1}<\ldots \;{<ℷ}_{\partial }\leq p}\left(1-{\left|-\left(\prod_{ℷ=1}^{\partial }\varphi_{j_{ℷ}}\left(x\right)\right)\right|}^{\frac{1}{\partial }}\right)\right) \end{array}\right)$$

$${\left(\underset{1\leq {ℷ}_{1}<\ldots \;{<ℷ}_{\partial }\leq p}{⨂}\frac{\left(\overset{\partial }{\underset{ℷ=1}{⨁}}\tau_{j_{ℷ}}\right)}{\partial }\right)}^{\frac{1}{C_{p-1}^{\partial }}}=\left(\begin{array}{c} {\left(\prod_{1\leq {ℷ}_{1}<\ldots \;{<ℷ}_{\partial }\leq p}\left(1-{\left(\prod_{ℷ=1}^{\partial }\left(1-\delta_{j_{ℷ}}\left(x\right)\right)\right)}^{\frac{1}{\partial }}\right)\right)}^{\frac{1}{C_{p-1}^{\partial }}}+\\ i{\left(\prod_{1\leq {ℷ}_{1}<\ldots \;{<ℷ}_{\partial }\leq p}\left(1-{\left(\prod_{ℷ=1}^{\partial }\left(1-\theta_{j_{ℷ}}\left(x\right)\right)\right)}^{\frac{1}{\partial }}\right)\right)}^{\frac{1}{C_{p-1}^{\partial }}},\\ -1+{\left(\prod_{1\leq {ℷ}_{1}<\ldots \;{<ℷ}_{\partial }\leq p}\left(1-{\left|-\left(\prod_{ℷ=1}^{\partial }\nu_{j_{ℷ}}\left(x\right)\right)\right|}^{\frac{1}{\partial }}\right)\right)}^{\frac{1}{C_{p-1}^{\partial }}}+\\ i\left(-1+{\left(\prod_{1\leq {ℷ}_{1}<\ldots \;{<ℷ}_{\partial }\leq p}\left(1-{\left|-\left(\prod_{ℷ=1}^{\partial }\varphi_{j_{ℷ}}\left(x\right)\right)\right|}^{\frac{1}{\partial }}\right)\right)}^{\frac{1}{C_{p-1}^{\partial }}}\right) \end{array}\right)$$

Property 7 *Let a family of BCFVs*$\tau_{j}=\left(\delta_{j}\left(x\right)+i\theta_{j}\left(x\right),\nu_{j}\left(x\right)+i\varphi_{j}\left(x\right)\right),j=1,2,\ldots ,n$*such that*$\tau_{j}=\tau$. *Then*:

$$BCFDHM\left(\tau_{1},\tau_{2},\ldots ,\tau_{n}\right)=\tau$$

Property 8 *Suppose that two sets of BCFVs*$\tau_{j}=\left(\delta_{j}\left(x\right)+i\theta_{j}\left(x\right),\nu_{j}\left(x\right)+i\varphi_{j}\left(x\right)\right)$*and*$\tau_{j}^{\prime }=\left(\delta_{j}^{\prime }\left(x\right)+i\theta_{j}^{\prime }\left(x\right),\nu_{j}^{\prime }\left(x\right)+i\varphi_{j}^{\prime }\left(x\right)\right),j=1,2,\ldots ,n$. *If*$\tau_{j}\leq \tau_{j}^{\prime }$*such that*$\delta_{j}\left(x\right)\leq \delta_{j}^{\prime }\left(x\right),\theta_{j}\left(x\right)\leq \theta_{j}^{\prime }\left(x\right)$*and*$\nu_{j}\left(x\right)\geq \nu_{j}^{\prime }\left(x\right),\varphi_{j}\left(x\right)\geq \varphi_{j}^{\prime }\left(x\right)$. *Then*:

$$BCFDHM\left(\tau_{1},\tau_{2},\ldots ,\tau_{n}\right)\leq BCFDHM\left(\tau_{1}^{\prime },\tau_{2}^{\prime },\ldots ,\tau_{n}^{\prime }\right)$$

Property 9 *Let a family of BCFVs*$\tau_{j}=\left(\delta_{j}\left(x\right)+i\theta_{j}\left(x\right),\nu_{j}\left(x\right)+i\varphi_{j}\left(x\right)\right),j=1,2,\ldots ,n$. *If*$\tau^{-}=\min\left(\tau_{1},\tau_{2},\ldots ,\tau_{n}\right)$*and*$\tau^{+}=\max\left(\tau_{1},\tau_{2},\ldots ,\tau_{n}\right)$. *Then*:

$$\tau^{-}\leq BCFDHM\left(\tau_{1},\tau_{2},\ldots ,\tau_{n}\right)\leq \tau^{+}$$

Definition 12 Let a family of BCFVs $\tau_{j}=\left(\delta_{j}\left(x\right)+i\theta_{j}\left(x\right),\nu_{j}\left(x\right)+i\varphi_{j}\left(x\right)\right),j=1,2,\ldots ,n$ with weight vector $w={\left(w_{1},w_{2},\ldots ,w_{n}\right)}^{T},w_{i}\in \left[0,1\right]$ and $\sum_{i=1}^{n}w_{i}=1$. The BCFWDHM operator is defined in Eq. (11), and we have:

(11) $${BCFWDHM}^{\left(\partial \right)}\left(\tau_{1},\tau_{2}.\;.\;.,\tau_{p}\right)=\left\{ \begin{array}{cc} {\left(\underset{1\leq {ℷ}_{1}<\ldots \;{<ℷ}_{\partial }\leq p\;}{⨂}\left(1-\sum_{ℷ=1}^{\partial }w_{i_{j}}\right)\left(\frac{\overset{\partial }{\underset{ℷ=1}{⨁}}\tau_{j_{ℷ}}}{\partial }\right)\right)}^{\frac{1}{C_{p-1}^{\partial }}} & 1\leq \partial <p\\ \overset{\partial }{\underset{ℷ=1}{⨁}}\tau_{j_{ℷ}}^{\frac{1-w_{i}}{p-1}} & \partial =p \end{array}\right.$$

Theorem 5 *Let a family of BCFVs*$\tau_{j}=\left(\delta_{j}\left(x\right)+i\theta_{j}\left(x\right),\nu_{j}\left(x\right)+i\varphi_{j}\left(x\right)\right),j=1,2,\ldots ,n$*with weight vector*$w={\left(w_{1},w_{2},\ldots ,w_{n}\right)}^{T},w_{i}\in \left[0,1\right]$*and*$\sum_{i=1}^{n}w_{i}=1$. *Then*, *the BCFWDHM operator is defined in the following* Eq. (12) *and we have*:

(12) $${BCFWDHM}^{\left(\partial \right)}\left(\tau_{1},\tau_{2}.\;.\;.,\tau_{p}\right)=\left(\begin{array}{c} {\left(\prod_{1\leq {ℷ}_{1}<\ldots \;{<ℷ}_{\partial }\leq p}\left(1-{\left({\left(\prod_{ℷ=1}^{\partial }\left(1-\delta_{j_{ℷ}}\left(x\right)\right)\right)}^{\frac{1}{\partial }}\right)}^{\left(1-\sum_{ℷ=1}^{\partial }w_{i_{j}}\right)}\right)\right)}^{\frac{1}{C_{p-1}^{\partial }}}+\\ i{\left(\prod_{1\leq {ℷ}_{1}<\ldots \;{<ℷ}_{\partial }\leq p}\left(1-{\left({\left(\prod_{ℷ=1}^{\partial }\left(1-\theta_{j_{ℷ}}\left(x\right)\right)\right)}^{\frac{1}{\partial }}\right)}^{\left(1-\sum_{ℷ=1}^{\partial }w_{i_{j}}\right)}\right)\right)}^{\frac{1}{C_{p-1}^{\partial }}},\\ -1+{\left(\prod_{1\leq {ℷ}_{1}<\ldots \;{<ℷ}_{\partial }\leq p}\left(1-{\left|-{\left|-\left(\prod_{ℷ=1}^{\partial }\nu_{j_{ℷ}}\left(x\right)\right)\right|}^{\frac{1}{\partial }}\right|}^{\left(1-\sum_{ℷ=1}^{\partial }w_{i_{j}}\right)}\right)\right)}^{\frac{1}{C_{p-1}^{\partial }}}+\\ i\left(-1+{\left(\prod_{1\leq {ℷ}_{1}<\ldots \;{<ℷ}_{\partial }\leq p}\left(1-{\left|-{\left|-\left(\prod_{ℷ=1}^{\partial }\varphi_{j_{ℷ}}\left(x\right)\right)\right|}^{\frac{1}{\partial }}\right|}^{\left(1-\sum_{ℷ=1}^{\partial }w_{i_{j}}\right)}\right)\right)}^{\frac{1}{C_{p-1}^{\partial }}}\right) \end{array}\right),1\leq \partial <p=\left(\begin{array}{c} 1-\prod_{ℷ=1}^{\partial }\left(1-{\left(\delta_{j_{ℷ}}\left(x\right)\right)}^{\frac{1-w_{i}}{p-1}}\right)+\\ i\left(1-\prod_{ℷ=1}^{\partial }\left(1-{\left(\theta_{j_{ℷ}}\left(x\right)\right)}^{\frac{1-w_{i}}{p-1}}\right)\right),\\ -\left(\prod_{ℷ=1}^{\partial }\left(-1+{\left(1+\nu_{j_{ℷ}}\left(x\right)\right)}^{\frac{1-w_{i}}{p-1}}\right)\right)+\\ i\left(-\prod_{ℷ=1}^{\partial }\left(-1+{\left(1+\varphi_{j_{ℷ}}\left(x\right)\right)}^{\frac{1-w_{i}}{p-1}}\right)\right) \end{array}\right),\partial =p$$

Proof Since a family of BCFVs $\tau_{j}=\left(\delta_{j}\left(x\right)+i\theta_{j}\left(x\right),\nu_{j}\left(x\right)+i\varphi_{j}\left(x\right)\right),j=1,2,\ldots ,n$ and stepwise prove also given as follows:

Case 1 Suppose that for 1≤∂<p.

$$\overset{\partial }{\underset{ℷ=1}{⨁}}\tau_{j_{ℷ}}=\left(\begin{array}{c} 1-\prod_{ℷ=1}^{\partial }\left(1-\delta_{j_{ℷ}}\left(x\right)\right)+i\left(1-\prod_{ℷ=1}^{\partial }\left(1-\theta_{j_{ℷ}}\left(x\right)\right)\right),\\ -\left(\prod_{ℷ=1}^{\partial }\nu_{j_{ℷ}}\left(x\right)\right)+i\left(-\left(\prod_{ℷ=1}^{\partial }\varphi_{j_{ℷ}}\left(x\right)\right)\right) \end{array}\right)$$

$$\left(\frac{\overset{\partial }{\underset{ℷ=1}{⨁}}\tau_{j_{ℷ}}}{\partial }\right)=\left(\begin{array}{c} 1-{\left(\prod_{ℷ=1}^{\partial }\left(1-\delta_{j_{ℷ}}\left(x\right)\right)\right)}^{\frac{1}{\partial }}+i\left(1-{\left(\prod_{ℷ=1}^{\partial }\left(1-\theta_{j_{ℷ}}\left(x\right)\right)\right)}^{\frac{1}{\partial }}\right),\\ -{\left|-\left(\prod_{ℷ=1}^{\partial }\nu_{j_{ℷ}}\left(x\right)\right)\right|}^{\frac{1}{\partial }}+i\left(-{\left|-\left(\prod_{ℷ=1}^{\partial }\varphi_{j_{ℷ}}\left(x\right)\right)\right|}^{\frac{1}{\partial }}\right) \end{array}\right)$$

$$\left(1-\sum_{ℷ=1}^{\partial }w_{i_{j}}\right)\left(\frac{\overset{\partial }{\underset{ℷ=1}{⨁}}\tau_{j_{ℷ}}}{\partial }\right)=\left(\begin{array}{c} 1-{\left({\left(\prod_{ℷ=1}^{\partial }\left(1-\delta_{j_{ℷ}}\left(x\right)\right)\right)}^{\frac{1}{\partial }}\right)}^{\left(1-\sum_{ℷ=1}^{\partial }w_{i_{j}}\right)}+\\ i\left(1-{\left({\left(\prod_{ℷ=1}^{\partial }\left(1-\theta_{j_{ℷ}}\left(x\right)\right)\right)}^{\frac{1}{\partial }}\right)}^{\left(1-\sum_{ℷ=1}^{\partial }w_{i_{j}}\right)}\right),\\ -{\left|-{\left|-\left(\prod_{ℷ=1}^{\partial }\nu_{j_{ℷ}}\left(x\right)\right)\right|}^{\frac{1}{\partial }}\right|}^{\left(1-\sum_{ℷ=1}^{\partial }w_{i_{j}}\right)}+\\ i\left(-{\left|-{\left|-\left(\prod_{ℷ=1}^{\partial }\varphi_{j_{ℷ}}\left(x\right)\right)\right|}^{\frac{1}{\partial }}\right|}^{\left(1-\sum_{ℷ=1}^{\partial }w_{i_{j}}\right)}\right) \end{array}\right)$$

$$\underset{1\leq {ℷ}_{1}<\ldots \;{<ℷ}_{\partial }\leq p\;}{⨂}\left(1-\sum_{ℷ=1}^{\partial }w_{i_{j}}\right)\left(\frac{\overset{\partial }{\underset{ℷ=1}{⨁}}\tau_{j_{ℷ}}}{\partial }\right)=\left(\begin{array}{c} \prod_{1\leq {ℷ}_{1}<\ldots \;{<ℷ}_{\partial }\leq p}\left(1-{\left({\left(\prod_{ℷ=1}^{\partial }\left(1-\delta_{j_{ℷ}}\left(x\right)\right)\right)}^{\frac{1}{\partial }}\right)}^{\left(1-\sum_{ℷ=1}^{\partial }w_{i_{j}}\right)}\right)+\\ i\prod_{1\leq {ℷ}_{1}<\ldots \;{<ℷ}_{\partial }\leq p}\left(1-{\left({\left(\prod_{ℷ=1}^{\partial }\left(1-\theta_{j_{ℷ}}\left(x\right)\right)\right)}^{\frac{1}{\partial }}\right)}^{\left(1-\sum_{ℷ=1}^{\partial }w_{i_{j}}\right)}\right)\\ -1+\prod_{1\leq {ℷ}_{1}<\ldots \;{<ℷ}_{\partial }\leq p}\left(1-{\left|-{\left|-\left(\prod_{ℷ=1}^{\partial }\nu_{j_{ℷ}}\left(x\right)\right)\right|}^{\frac{1}{\partial }}\right|}^{\left(1-\sum_{ℷ=1}^{\partial }w_{i_{j}}\right)}\right)+\\ i\left(-1+\prod_{1\leq {ℷ}_{1}<\ldots \;{<ℷ}_{\partial }\leq p}\left(1-{\left|-{\left|-\left(\prod_{ℷ=1}^{\partial }\varphi_{j_{ℷ}}\left(x\right)\right)\right|}^{\frac{1}{\partial }}\right|}^{\left(1-\sum_{ℷ=1}^{\partial }w_{i_{j}}\right)}\right)\right) \end{array}\right)$$

$${\left(\underset{1\leq {ℷ}_{1}<\ldots \;{<ℷ}_{\partial }\leq p\;}{⨂}\left(1-\sum_{ℷ=1}^{\partial }w_{i_{j}}\right)\left(\frac{\overset{\partial }{\underset{ℷ=1}{⨁}}\tau_{j_{ℷ}}}{\partial }\right)\right)}^{\frac{1}{C_{p-1}^{\partial }}}=\left(\begin{array}{c} {\left(\prod_{1\leq {ℷ}_{1}<\ldots \;{<ℷ}_{\partial }\leq p}\left(1-{\left({\left(\prod_{ℷ=1}^{\partial }\left(1-\delta_{j_{ℷ}}\left(x\right)\right)\right)}^{\frac{1}{\partial }}\right)}^{\left(1-\sum_{ℷ=1}^{\partial }w_{i_{j}}\right)}\right)\right)}^{\frac{1}{C_{p-1}^{\partial }}}+\\ i{\left(\prod_{1\leq {ℷ}_{1}<\ldots \;{<ℷ}_{\partial }\leq p}\left(1-{\left({\left(\prod_{ℷ=1}^{\partial }\left(1-\theta_{j_{ℷ}}\left(x\right)\right)\right)}^{\frac{1}{\partial }}\right)}^{\left(1-\sum_{ℷ=1}^{\partial }w_{i_{j}}\right)}\right)\right)}^{\frac{1}{C_{p-1}^{\partial }}},\\ -1+{\left(\prod_{1\leq {ℷ}_{1}<\ldots \;{<ℷ}_{\partial }\leq p}\left(1-{\left|-{\left|-\left(\prod_{ℷ=1}^{\partial }\nu_{j_{ℷ}}\left(x\right)\right)\right|}^{\frac{1}{\partial }}\right|}^{\left(1-\sum_{ℷ=1}^{\partial }w_{i_{j}}\right)}\right)\right)}^{\frac{1}{C_{p-1}^{\partial }}}+\\ i\left(-1+{\left(\prod_{1\leq {ℷ}_{1}<\ldots \;{<ℷ}_{\partial }\leq p}\left(1-{\left|-{\left|-\left(\prod_{ℷ=1}^{\partial }\varphi_{j_{ℷ}}\left(x\right)\right)\right|}^{\frac{1}{\partial }}\right|}^{\left(1-\sum_{ℷ=1}^{\partial }w_{i_{j}}\right)}\right)\right)}^{\frac{1}{C_{p-1}^{\partial }}}\right) \end{array}\right)$$

Case 2 For ∂=p.

$$\tau_{j_{ℷ}}^{\frac{1-w_{i}}{p-1}}=\left(\begin{array}{c} {\left(\delta_{j_{ℷ}}\left(x\right)\right)}^{\frac{1-w_{i}}{p-1}}+i{\left(\theta_{j_{ℷ}}\left(x\right)\right)}^{\frac{1-w_{i}}{p-1}},\\ -1+{\left(1+\nu_{j_{ℷ}}\left(x\right)\right)}^{\frac{1-w_{i}}{p-1}}+i\left(-1+{\left(1+\varphi_{j_{ℷ}}\left(x\right)\right)}^{\frac{1-w_{i}}{p-1}}\right) \end{array}\right)$$

$$\overset{\partial }{\underset{ℷ=1}{⨁}}\tau_{j_{ℷ}}^{\frac{1-w_{i}}{p-1}}=\left(\begin{array}{c} 1-\prod_{ℷ=1}^{\partial }\left(1-{\left(\delta_{j_{ℷ}}\left(x\right)\right)}^{\frac{1-w_{i}}{p-1}}\right)+\\ i\left(1-\prod_{ℷ=1}^{\partial }\left(1-{\left(\theta_{j_{ℷ}}\left(x\right)\right)}^{\frac{1-w_{i}}{p-1}}\right)\right),\\ -\left(\prod_{ℷ=1}^{\partial }\left(-1+{\left(1+\nu_{j_{ℷ}}\left(x\right)\right)}^{\frac{1-w_{i}}{p-1}}\right)\right)+\\ i\left(-\prod_{ℷ=1}^{\partial }\left(-1+{\left(1+\varphi_{j_{ℷ}}\left(x\right)\right)}^{\frac{1-w_{i}}{p-1}}\right)\right) \end{array}\right)$$

Property 10 *Let a family of BCFVs*$\tau_{j}=\left(\delta_{j}\left(x\right)+i\theta_{j}\left(x\right),\nu_{j}\left(x\right)+i\varphi_{j}\left(x\right)\right),j=1,2,\ldots ,n$*such that*$\tau_{j}=\tau$. *Then*:

$$BCFWDHM\left(\tau_{1},\tau_{2},\ldots ,\tau_{n}\right)=\tau$$

Property 11 *Suppose that two sets of BCFVs*$\tau_{j}=\left(\delta_{j}\left(x\right)+i\theta_{j}\left(x\right),\nu_{j}\left(x\right)+i\varphi_{j}\left(x\right)\right)$*and*$\tau_{j}^{\prime }=\left(\delta_{j}^{\prime }\left(x\right)+i\theta_{j}^{\prime }\left(x\right),\nu_{j}^{\prime }\left(x\right)+i\varphi_{j}^{\prime }\left(x\right)\right),j=1,2,\ldots ,n$. *If*$\tau_{j}\leq \tau_{j}^{\prime }$*such that*$\delta_{j}\left(x\right)\leq \delta_{j}^{\prime }\left(x\right),\theta_{j}\left(x\right)\leq \theta_{j}^{\prime }\left(x\right)$*and*$\nu_{j}\left(x\right)\geq \nu_{j}^{\prime }\left(x\right),\varphi_{j}\left(x\right)\geq \varphi_{j}^{\prime }\left(x\right)$. *Then*:

$$BCFWDHM\left(\tau_{1},\tau_{2},\ldots ,\tau_{n}\right)\leq BCFWDHM\left(\tau_{1}^{\prime },\tau_{2}^{\prime },\ldots ,\tau_{n}^{\prime }\right)$$

Property 12 *Let a family of BCFVs*$\tau_{j}=\left(\delta_{j}\left(x\right)+i\theta_{j}\left(x\right),\nu_{j}\left(x\right)+i\varphi_{j}\left(x\right)\right),j=1,2,\ldots ,n$. *If*$\tau^{-}=\min\left(\tau_{1},\tau_{2},\ldots ,\tau_{n}\right)$*and*$\tau^{+}=\max\left(\tau_{1},\tau_{2},\ldots ,\tau_{n}\right)$. *Then*:

$$\tau^{-}\leq BCFWDHM\left(\tau_{1},\tau_{2},\ldots ,\tau_{n}\right)\leq \tau^{+}$$

## 5 Evaluation process with decision-making problem and derived approaches

The uniqueness of this section is that it presents an innovative approach to the MADM problem to evaluate an ideal solution under some specific characteristics or attributes. The main theme of this strategy is to identify kinds of attributes, organize fuzzy information about any object, aggregate collected fuzzy information, and investigate the best optimal options based on the ranking of score values of all individuals. To serve this purpose, consider a family of alternative $\left(A_{1},A_{2},\ldots ,A_{n}\right)$ and a set of attributes $\left(L_{1},L_{2},\ldots ,L_{m}\right)$. To proceed decision analysis process, the decision-maker assigns some particular weight $\left(w_{1},w_{2},\ldots ,w_{m}\right)$ to each attribute such that $w_{j}>0$ and $\sum_{j=1}^{m}w_{j}=1$. The decision-maker expresses their own opinion in the form of BCFVs shown in a decision matrix. Each alternative or individual has one or more types of attributes such as beneficial and non-beneficial. The evaluation and aggregation process are done using the following steps of an algorithm for the MADM problem.

Step 1 The decision-maker accumulates BCF information in the form of alternatives and attributes, which are demonstrated in a decision matrix.

Step 2 We noted that different types of attributes may be involved in each experimental case study. Before the aggregation process, all attributes should be the same type by using the following expression:

$$D=\left\{ \begin{array}{cc} \left(\delta_{j\iota }\left(x\right)+i\theta_{j\iota }\left(x\right),\nu_{j\iota }\left(x\right)+i\varphi_{j\iota }\left(x\right)\right) & benefit\;type\\ \left({1-\delta }_{j\iota }\left(x\right)+i\left(1-\theta_{j\iota }\left(x\right)\right),-1-\nu_{j\iota }\left(x\right)+i\left(-1-\varphi_{j\iota }\left(x\right)\right)\right) & non-beneficial\;type \end{array}\right.$$

Step 3 Applied derived approaches of the BCFWHM and BCFWDHM operators.

Step 4 Compute score values corresponding to each alternative. If the computed results of the score function are equal, then we use the accuracy function for evaluating the best optimal option based on Definition 4.

**Fig. 1:**
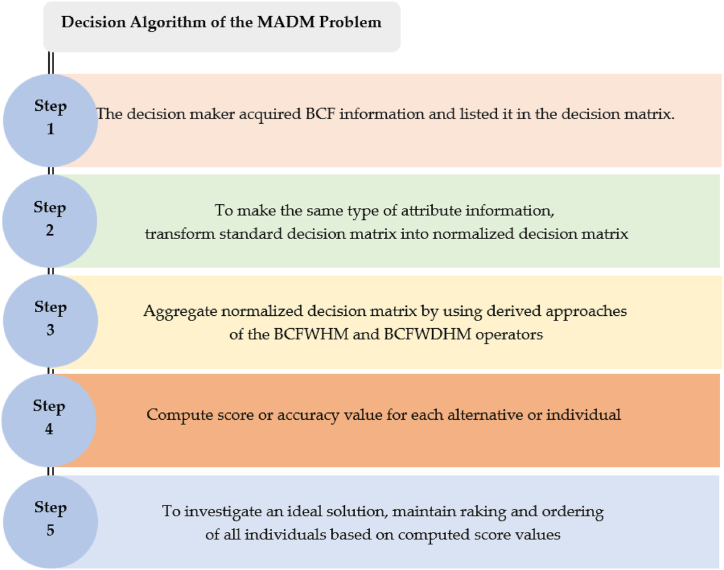
shows the steps of the decision algorithm of the MADM problem.

Step 5 To demonstrate a desirable optimal option, we need the ranking and ordering of score values of all alternatives. We also demonstrate the steps of the decision algorithm in Fig. 1.

### 5.1 Experimental case study

An individual investor might attain their financial and investing objectives by following a set of concepts referred to as investment strategies. An investor uses this strategy to inform their judgments about goals, risk tolerance, and future financial needs. Strategies for investing might range from extremely cautious ones to quite aggressive ones. Investment strategies that lean towards conservatism use secure assets with steady returns and minimal risk. A highly aggressive investment strategy involves taking on hazardous assets, including trash bonds, equities, and options, in order to maximize returns.

The significance of best investment ideas and strategies lies in their ability to help individuals and businesses optimize returns, manage risks, and work towards achieving specific financial goals. The best investment ideas and strategies are essential for individuals and businesses to navigate the complex world of finance. These strategies provide a roadmap for achieving financial goals, managing risks, and ultimately securing a more prosperous future. Regular reviews and adjustments to these strategies are crucial to adapt to changing circumstances and ensure continued effectiveness. Numerous research scholars discussed many dominant investment plans under particular characteristics or features with the help of decision analysis. For example, Ullah et al. [65] evaluated a suitable investment policy based on a decision algorithm of the MADM problem. Wells [66] discussed some viable strategies for understanding the ups and downs of business realities. We also studied different suggestions and recommendations in Refs. [[67], [68], [69]].

In this experimental case study, we explore some reliable investment plans for the growth of businesses and wealth accumulation. To serve this purpose, consider there are five different investment opportunities and plans discussed as follows.

Real Estate Investment Trusts ${Ѧ}_{1}$
Rental housing ${Ѧ}_{2}$
Electric Vehicles (EVs) and Clean Transportation ${Ѧ}_{3}$
Cryptocurrencies ${Ѧ}_{4}$
Bonds (Long-term corporate bond funds) ${Ѧ}_{5}$

By utilizing derived mathematical approaches and decision algorithm of the MADM problem, we investigate a reliable opportunity under the following characteristics or attributes.

Risk analysis ${Њ}_{1}$
Time horizon ${Њ}_{2}$
Tax implications and scalability ${Њ}_{3}$
Liquidity and market demand ${Њ}_{4}$

To evaluate a suitable investment opportunity, we assumed the degree of weight (0.30,0.35,0.15,0.20) for each attribute or characteristic in this experimental case study. So, we can also investigate the weight of criteria using different methods or techniques. The above-discussed experimental case study is investigated considering the derived research work and mathematical approaches of the BCFWHM and BCFWDHM operators.

**Step 1:** The decision maker acquires human information in the form of BCFVs and arranges them in a decision matrix of Table 1.

**Table 1:** Arranges BCF information in a decision matrix $D_{1}$.

Alternatives	${Њ}_{1}$	${Њ}_{2}$	${Њ}_{3}$	${Њ}_{4}$
${Ѧ}_{1}$	$\left(\begin{array}{c} 0.24+i0.31\text{,}\\ -0.19-i0.15 \end{array}\right)$	$\left(\begin{array}{c} 0.22+i0.34\text{,}\\ -0.11-i0.17 \end{array}\right)$	$\left(\begin{array}{c} 0.25+i0.43\text{,}\\ -0.16-i0.24 \end{array}\right)$	$\left(\begin{array}{c} 0.33+i0.39\text{,}\\ -0.17-i0.18 \end{array}\right)$
${Ѧ}_{2}$	$\left(\begin{array}{c} 0.19+i0.71\text{,}\\ -0.45-i0.27 \end{array}\right)$	$\left(\begin{array}{c} 0.31+i0.54\text{,}\\ -0.32-i0.57 \end{array}\right)$	$\left(\begin{array}{c} 0.26+i0.69\text{,}\\ -0.18-i0.34 \end{array}\right)$	$\left(\begin{array}{c} 0.43+i0.45\text{,}\\ -0.32-i0.36 \end{array}\right)$
${Ѧ}_{3}$	$\left(\begin{array}{c} 0.53+i0.61\text{,}\\ -0.46-i0.57 \end{array}\right)$	$\left(\begin{array}{c} 0.53+i0.65\text{,}\\ -0.38-i0.59 \end{array}\right)$	$\left(\begin{array}{c} 0.44+i0.49\text{,}\\ -0.38-i0.56 \end{array}\right)$	$\left(\begin{array}{c} 0.39+i0.56\text{,}\\ -0.38-i0.59 \end{array}\right)$
${Ѧ}_{4}$	$\left(\begin{array}{c} 0.27+i0.7\text{,}\\ -0.38-i0.43 \end{array}\right)$	$\left(\begin{array}{c} 0.27+i0.43\text{,}\\ -0.44-i0.48 \end{array}\right)$	$\left(\begin{array}{c} 0.21+i0.51\text{,}\\ -0.28-i0.69 \end{array}\right)$	$\left(\begin{array}{c} 0.46+i0.53\text{,}\\ -0.34-i0.47 \end{array}\right)$
${Ѧ}_{5}$	$\left(\begin{array}{c} 0.29+i0.45\text{,}\\ -0.18-i0.34 \end{array}\right)$	$\left(\begin{array}{c} 0.49+i0.5\text{,}\\ -0.18-i0.69 \end{array}\right)$	$\left(\begin{array}{c} 0.22+i0.53\text{,}\\ -0.31-i0.61 \end{array}\right)$	$\left(\begin{array}{c} 0.45+i0.65\text{,}\\ -0.26-i0.65 \end{array}\right)$

**Step 2:**Table 1 contains only one type of beneficial information, so it is unnecessary to normalize the given decision matrix into a normalized decision matrix.

**Step 3:** By utilizing derived mathematical approaches of the BCFWHM and BCFWDHM operators at a fixed value of ∂=2, aggregate information of different preferences or attributes corresponding to each alternative and computed outcomes are drawn in Table 2.

**Table 2:** Aggregated outcomes by the BCFWHM and BCFWDHM operators at a fixed value of ∂=2.

BCFWHM	BCFWDHM
$\left(\begin{array}{c} 0.2607+i0.3704\text{,}\\ -0.1582-i0.1873 \end{array}\right)$	$\left(\begin{array}{c} 0.0188+i0.0404\text{,}\\ -0.6433-i0.6814 \end{array}\right)$
$\left(\begin{array}{c} 0.2929+i0.5965\text{,}\\ -0.3106-i0.3789 \end{array}\right)$	$\left(\begin{array}{c} 0.0252+i0.1334\text{,}\\ -0.8104-i0.8564 \end{array}\right)$
$\left(\begin{array}{c} 0.4657+i0.5706\text{,}\\ -0.3988-i0.5769 \end{array}\right)$	$\left(\begin{array}{c} 0.0732+i0.1203\text{,}\\ -0.8692-i0.9443 \end{array}\right)$
$\left(\begin{array}{c} 0.2954+i0.5406\text{,}\\ -0.3542-i0.5302 \end{array}\right)$	$\left(\begin{array}{c} 0.0261+i0.1039\text{,}\\ -0.8446-i0.9219 \end{array}\right)$
$\left(\begin{array}{c} 0.3473+i0.5353\text{,}\\ -0.2374-i0.5826 \end{array}\right)$	$\left(\begin{array}{c} 0.0398+i0.0975\text{,}\\ -0.7336-i0.9409 \end{array}\right)$

**Step 4:** By utilizing Definition 4, compute the score values of all individuals. Table 3 illustrates the score values of all alternatives or individuals.

**Table 3:** Computed score values of all alternatives.

Aggregation operators	$H\left({Ѧ}_{1}\right)$	$H\left({Ѧ}_{2}\right)$	$H\left({Ѧ}_{3}\right)$	$H\left({Ѧ}_{4}\right)$	$H\left({Ѧ}_{5}\right)$	Ranking of Alternatives
BCFWHM	0.5714	0.5500	0.5151	0.4879	0.5156	$D_{1}≻D_{2}≻D_{5}≻D_{3}≻D_{4}$
BCFWDHM	0.1836	0.1230	0.0950	0.0909	0.1157	$D_{1}≻D_{2}≻D_{5}≻D_{3}≻D_{4}$

**Fig. 2:**
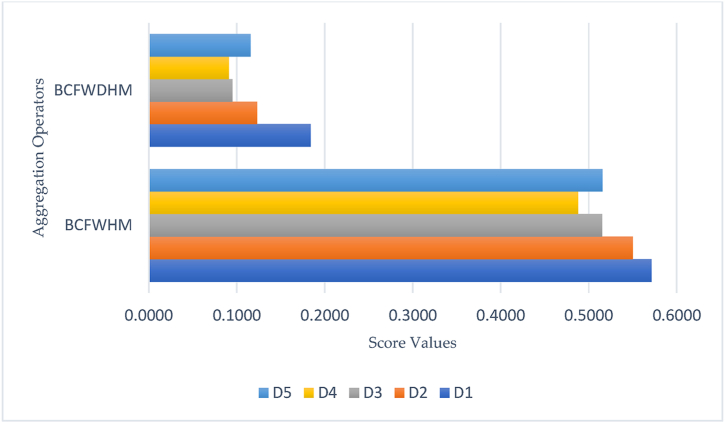
Results of BCFWHM and BCFWDHM operators.

**Step 5:** To investigate an ideal solution for an alternative or individual, the ranking and ordering of all score values corresponding to each alternative are also managed in Table 3. Fig. 2 also offers a better understanding to the reader about the aggregated outcomes and results of all options. Geometrical representations are dominant components of the decision analysis and aggregation process, and the different colors in Fig. 2 show the reliability of different alternatives or individuals.

### 5.2 Sensitive analysis

In this subsection, the authors demonstrate the applicability and robustness of derived approaches by utilizing an algorithm of the MADM problem. By employing the different values ∂ in the decision-making process, decision-makers aggregate the results of score values and their corresponding ranking of alternatives. The uniqueness of this technique is that it shows intensity and consistency during decision analysis.

**Fig. 3:**
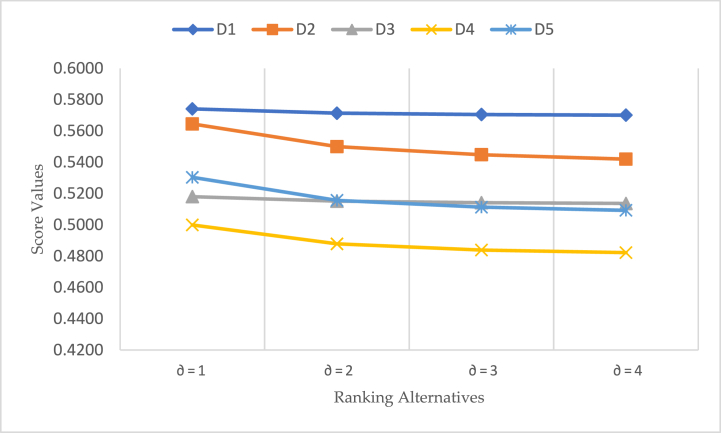
Results score value by the BCFWHM operator at different values of ∂.

Now, we illustrate aggregated outcomes by the BCFWHM operator at different values ∂ in the MADM problem. Aggregated outcomes of the score values and ranking of alternatives are shown in Table 4. After analysis of computed score values, it is clear that the ranking of alternative $D_{1}≻D_{2}≻D_{5}≻D_{3}≻D_{4}$ unchanged at different values ∂*.* Table 4 shows the aggregated outcomes of the BCFWHM operator. Geometrical shape is another component of the MADM problem, which provides a clear understanding of the decision support system. Fig. 3 shows the computed results by the BCFWHM operator, which is listed in Table 4.

**Table 4:** Investigated score values and their ranking by the BCFWHM operator at different values ∂.

Parametric value	$H\left({Ѧ}_{1}\right)$	$H\left({Ѧ}_{2}\right)$	$H\left({Ѧ}_{3}\right)$	$H\left({Ѧ}_{4}\right)$	$H\left({Ѧ}_{5}\right)$	Ranking of Alternatives
∂=1	0.5741	0.5645	0.5180	0.4999	0.5304	$D_{1}≻D_{2}≻D_{5}≻D_{3}≻D_{4}$
∂=2	0.5714	0.5500	0.5151	0.4879	0.5156	$D_{1}≻D_{2}≻D_{5}≻D_{3}≻D_{4}$
∂=3	0.5705	0.5448	0.5141	0.4839	0.5113	$D_{1}≻D_{2}≻D_{3}≻D_{5}≻D_{4}$
∂=4	0.5701	0.5420	0.5137	0.4823	0.5093	$D_{1}≻D_{2}≻D_{3}≻D_{5}≻D_{4}$

**Fig. 4:**
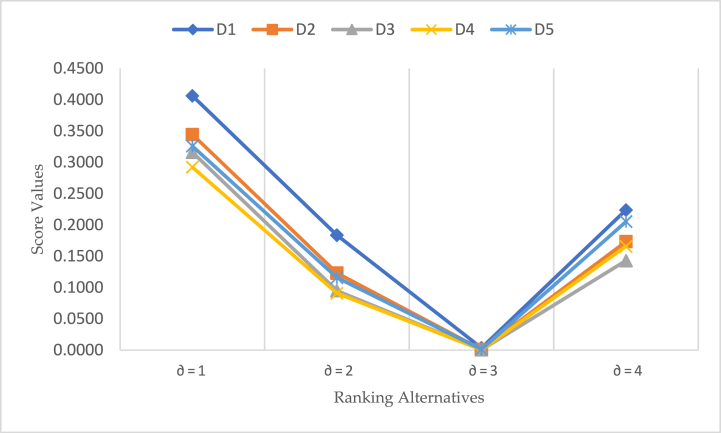
Results score value by the BCFWDHM operator at different values of ∂.

Table 5 also presents the investigated results of score values by the BCFDWHM operator at different values ∂. Ranking of alternatives $D_{1}≻D_{2}≻D_{5}≻D_{3}≻D_{4}$ and $D_{1}≻D_{5}≻D_{2}≻D_{4}≻D_{3}$ for the value ∂=1,2 and ∂=3,4 respectively. This uniqueness of the constant raking of alternatives shows the reliability and consistency of derived approaches. Fig. 4 explore the reliability of investigated results by the BCFWDHM operators.

**Table 5:** Investigated score values and their ranking by the BCFWDHM operator at different values ∂.

Parametric value	$H\left({Ѧ}_{1}\right)$	$H\left({Ѧ}_{2}\right)$	$H\left({Ѧ}_{3}\right)$	$H\left({Ѧ}_{4}\right)$	$H\left({Ѧ}_{5}\right)$	Ranking of Alternatives
∂=1	0.4062	0.3444	0.3159	0.2922	0.3258	$D_{1}≻D_{2}≻D_{5}≻D_{3}≻D_{4}$
∂=2	0.1836	0.1230	0.0950	0.0909	0.1157	$D_{1}≻D_{2}≻D_{5}≻D_{3}≻D_{4}$
∂=3	0.0035	0.0011	0.0005	0.0006	0.0012	$D_{1}≻D_{5}≻D_{2}≻D_{4}≻D_{3}$
∂=4	0.2237	0.1734	0.1429	0.1657	0.2053	$D_{1}≻D_{5}≻D_{2}≻D_{4}≻D_{3}$

## 6 Comparative study

In this discussion, the authors demonstrate the robustness and validity of the diagnosed decision algorithm of the MADM problem based on derived research work and strategies. For this purpose, we studied many existing realistic mathematical approaches and invented theories [32,64,[70], [71], [72], [73], [74], [75]]. Xu [70] investigated a series of aggregation operators that take into account the theory of arithmetic and geometric mean operators. Hussain et al. [71] classified important properties of HM operators to develop new methodologies in the light of complex intuitionistic fuzzy information. Wei et al. [72] illustrated the characteristics of Hamacher operators to investigate an ideal solution under considering specific criteria or attributes with bipolar fuzzy information. Mahmood et al. [64] developed some dominant strategies of weighted average and weighted geometric operators in the presence of the BCF domain. Mahmood and Rehman [73] also proposed aggregation operators of Dombi t-norm and t-conorm in the light of BCF theory. Mahmood et al. [32] modified the theory of Aczel Alsina operations to derive mathematical approaches that take into account BCF information. We noticed that discussed mathematical terminologies [[70], [71], [72],^74^,^75^] fail due to their narrow and limited structure. Table 6 explores all investigated results by utilizing the decision algorithm of the MADM problem and mathematical methodologies.

**Table 6:** shows aggregated outcomes by the existing approaches.

Aggregation Operators	$H\left({Ѧ}_{1}\right)$	$H\left({Ѧ}_{2}\right)$	$H\left({Ѧ}_{3}\right)$	$H\left({Ѧ}_{4}\right)$	$H\left({Ѧ}_{5}\right)$	Ranking of Alternatives
Xu [70]	˟	˟	˟	˟	˟	✗✗✗✗✗
Hussain et al. [71]	˟	˟	˟	˟	˟	✗✗✗✗✗
Wei et al. [72]	˟	˟	˟	˟	˟	✗✗✗✗✗
Gurmani et al. [74]	˟	˟	˟	˟	˟	✗✗✗✗✗
Hussain et al. [75]	˟	˟	˟	˟	˟	✗✗✗✗✗
BCFWHM (Currently)	0.5714	0.5500	0.5151	0.4879	0.5156	$D_{1}≻D_{2}≻D_{5}≻D_{3}≻D_{4}$
BCFWDHM (Currently)	0.1836	0.1230	0.0950	0.0909	0.1157	$D_{1}≻D_{2}≻D_{5}≻D_{3}≻D_{4}$
BCFAAWA [32]	0.3467	0.3004	0.2800	0.2649	0.2990	$D_{1}≻D_{2}≻D_{5}≻D_{3}≻D_{4}$
BCFWA [64]	0.5714	0.5487	0.5273	0.5001	0.5411	$D_{1}≻D_{2}≻D_{5}≻D_{3}≻D_{4}$
BCFWG [64]	0.5676	0.5257	0.5233	0.4844	0.5188	$D_{1}≻D_{2}≻D_{5}≻D_{3}≻D_{4}$
BCFDWA [73]	0.3774	0.3837	0.2968	0.3302	0.3699	$D_{2}≻D_{1}≻D_{5}≻D_{4}≻D_{3}$
BCFDWG [73]	0.3691	0.3707	0.2931	0.3189	0.3606	$D_{2}≻D_{1}≻D_{5}≻D_{4}≻D_{3}$

**Fig. 5:**
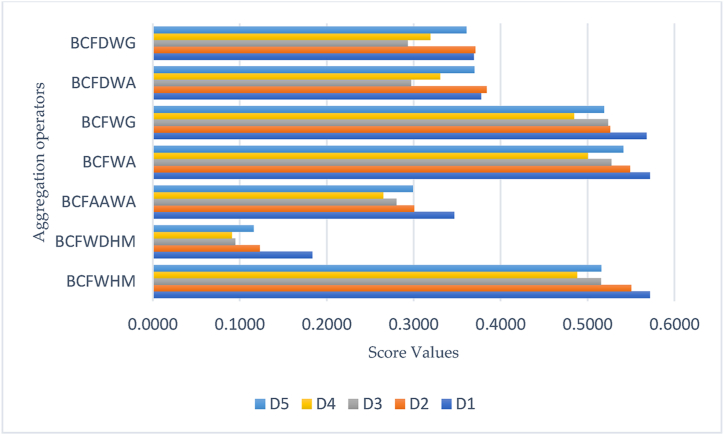
Geometrical representation shows aggregated outcomes by the existing approaches.

To understand the structural behavior of aggregated outcomes and the reliability of diagnosed operators, we explore all compute results in a bar chart in Fig. 5. The geometrical representations are an effective component of the decision-making process and provide readers with a clear understanding of invented results. However, diagnosed operators are more reliable and authentic than previously developed mathematical approaches.

## Conclusion

The main feature of diagnosed research work and proposed mathematical strategies is to manage uncertain and ambiguous information about human opinion. An innovative decision algorithm for the MADM problem is also illustrated to resolve complicated real-life applications and different experimental case studies. In this manuscript, authors explored the dominant concepts of HM and DHM operators to describe correlation among different input arguments under the system of BCF environment. By generalizing the theory of product t-norm and the probabilistic sum of t-conorm, we developed a family of realistic mathematical approaches based on BCF information, including BCFHM, BCFWHM, BCFDHM, and BCFWDHM operators. Some notable characteristics and properties are also described to show the validity and robustness of invented approaches. Additionally, an algorithm for the MADM problem is established to resolve complicated genuine real-life applications by considering derived research work and mathematical methodologies. A numerical example is studied to evaluate an ideal solution of the alternatives and also shows the compatibility of derived mathematical methodologies. The advantages and consistency of invented research work are also explored with geometrical representations. Finally, we demonstrated the supremacy and effectiveness of diagnosed research work with the comparison technique of existing research work.

We noted that diagnosed mathematical approaches have many advantages but still have some limitations and drawbacks. Firstly, derived approaches are only applicable for knowing the degree of weights to each attribute. Secondly, it has a limited and narrow structure due to expert preferences. However, if an expert wants to acquire an independent degree of information, then this developed research work needs to be modified. In the coming future, we will generalize our invented research work in different fuzzy domains, such as bipolar picture fuzzy theory [76], T-spherical fuzzy hypersoft sets [77], and spherical and t-spherical fuzzy environments [78]. We can also utilize our presented research work to resolve different advanced decision analysis methods, such as the TOPSIS method [79], the WASPAS method [80], the EDAS method [81], the VIKOR method [82] and the quasirung fuzzy sets [83,84].

## Data availability

The data will be available on reasonable request to the corresponding author.

## CRediT authorship contribution statement

**Zhuoan Zhao:** Validation, Investigation, Formal analysis, Data curation. **Abrar Hussain:** Writing – review & editing, Writing – original draft, Investigation, Conceptualization. **Nan Zhang:** Software, Resources, Funding acquisition, Data curation, Conceptualization. **Kifayat Ullah:** Visualization, Validation, Supervision, Investigation, Conceptualization. **Shi Yin:** Software, Resources, Project administration, Funding acquisition, Formal analysis. **Amrullah Awsar:** Validation, Project administration, Funding acquisition, Data curation, Conceptualization. **Salah M. El-Bahy:** Writing – original draft, Visualization, Validation, Formal analysis.

## Declaration of competing interest

The authors declare that they have no known competing financial interests or personal relationships that could have appeared to influence the work reported in this paper.
